# Natural variations of *SLG1* confer high-temperature tolerance in *indica* rice

**DOI:** 10.1038/s41467-020-19320-9

**Published:** 2020-10-28

**Authors:** Yufang Xu, Li Zhang, Shujun Ou, Ruci Wang, Yueming Wang, Chengcai Chu, Shanguo Yao

**Affiliations:** 1 State Key Laboratory of Plant Genomics, Institute of Genetics and Developmental Biology, the Innovative Academy of Seed Design, Chinese Academy of Sciences, Beijing, 100101 China; 2 College of Advanced Agricultural Sciences, University of Chinese Academy of Sciences, Beijing, 100039 China; 3 Department of Ecology, Evolution, and Organismal Biology, Iowa State University, Ames, IA 50011 USA

**Keywords:** Agricultural genetics, Agricultural genetics, Heat

## Abstract

With global warming and climate change, breeding crop plants tolerant to high-temperature stress is of immense significance. tRNA 2-thiolation is a highly conserved form of tRNA modification among living organisms. Here, we report the identification of *SLG1* (*Slender Guy 1*), which encodes the cytosolic tRNA 2-thiolation protein 2 (RCTU2) in rice. *SLG1* plays a key role in the response of rice plants to high-temperature stress at both seedling and reproductive stages. Dysfunction of *SLG1* results in plants with thermosensitive phenotype, while overexpression of *SLG1* enhances the tolerance of plants to high temperature. *SLG1* is differentiated between the two Asian cultivated rice subspecies, *indica* and *japonica*, and the variations at both promoter and coding regions lead to an increased level of thiolated tRNA and enhanced thermotolerance of *indica* rice varieties. Our results demonstrate that the allelic differentiation of *SLG1* confers *indica* rice to high-temperature tolerance, and tRNA thiolation pathway might be a potential target in the next generation rice breeding for the warming globe.

## Abstract (web summary)

Understanding the mechanism of high-temperature tolerance will help to breed crops adaptive to warming climate. Here, the authors show *SLG1*, a cytosolic tRNA 2-thiolation protein 2 encoding gene, is differentiated between the two Asian cultivated rice subspecies and confers high temperature tolerance of *indica* rice.

## Introduction

Rice (*Oryza sativa* L.) is an important food crop for over half of the world’s population. The yield and quality of rice are often reduced by high-temperature stress^1,2^. Particularly, high temperature at the heading stage would cause premature degradation of anther tapetum, obstruction of pollen-tube elongation, abortion of pollen mother cells, decrease of pollen viability, and finally leading to reduction of spikelet fertility^3–5^. The high-temperature stress at the grain filling stage would result in poor milling quality, decreased grain weight and gel consistency, deteriorated starch structure, and increased chalkiness rate^6,7^. The damage of high-temperature stress to rice production would be further aggravated with climate change and global warming^2^.

Rice has adapted to different environmental conditions. The *TT1* gene isolated from African rice (*O. glaberrima* Steud.) has been shown to contribute to the adaptation of rice plants under distinct climatic temperature^8^. For Asian cultivated rice, there are two genetically distinct subspecies, *indica* and *japonica*. *indica* rice is mainly distributed in tropical and subtropical regions, while *japonica* rice is mainly in temperate and cooler regions. Apparently, typical *indica* and *japonica* cultivars have been locally adapted to different environmental temperatures during natural and artificial selection^9^. For example, it has been shown that *COLD1*^*jap*^ and *bZIP73*^*jap*^, the selected natural variants during *japonica* domestication, can partially explain why japonica has stronger cold tolerance than *indica*^10–12^; distinct haplotypes of *CTB4a* have also been artificially selected and contributed to cold tolerance at the booting stage in *japonica* rice^13^. However, the molecular basis underlying thermotolerance of *indica* rice is still poorly understood.

A recent analysis with the rice *aet1* mutant revealed the critical function of tRNA modification in the response to high temperature^14^. As the indispensable participants in protein synthesis, tRNAs undergo abundant post-transcriptional modifications in both prokaryotes and eukaryotes, which are crucial for all the core aspects of tRNA functions including stress response^15–17^. As an important modification form, tRNA 2-thiolation refers to the thiolation on position 2 (s^2^) of the tRNA wobble uridine (U_34_), which is often accompanied by the methoxycarbonyl methylation on position 5 (mcm^5^)^18,19^. mcm^5^s^2^U_34_ is exclusively found in three cytosolic tRNAs of tK^UUU^, tE^UUC^, and tQ^UUG^, and the s^2^ modification in yeast (*Saccharomyces cerevisiae*) has been shown to depend on the URM1 pathway^20–22^. In this pathway, the C-terminal rhodanese-like domain of ubiquitin activating 4 enzyme (Uba4) provides sulfur to the ubiquitin-related modifier (Urm1), thereby forms a thiocarboxylated Urm1 (Urm1-COSH). And after the reductive cleavage of the acyl disulfide bond between Uba4 and Urm1, Urm1-COSH gets released to donate the activated sulfur for insertion into the target tRNA uridine facilitated by two cytosolic tRNA 2-thiolation proteins, Ncs6/CTU1 and Ncs2/CTU2^20,22,23^, which catalyze the last step of the thiolation process by forming a functional complex^24^.

A number of evidences showed that tRNA 2-thiolation in U_34_ has important roles in decoding and translation, and its defectiveness leads to various diseases in humans and pleotropic phenotypes in yeast^21,25,26^. In yeast, lack of the U_34_ thiolation results in protein misfolding and aggregation^27^, inability to maintain metabolic homeostasis^15,28^, defects in invasive growth^29^, and hypersensitivity to various abiotic stresses^30,31^. In particular, many studies suggested the importance of the U_34_ thiolation in heat stress response. In yeast, the budding ability and propagation speed of the single and double mutants of *ctu1/ctu2* are significantly lower than that of the wild-type strain under elevated temperature^24^. And, multicopy expression of tRNA^LYS^, tRNA^GLU^, or a combination of both can rescue the growth defect of the *ctu1* mutant strain^24^. Furthermore, dysfunction of the *CTU1* homologs in archaea and *Caenorhabditis elegans* also causes thermosensitive phenotype^24,32^. Interestingly, a recent study compared the U_34_ thiolation profile from evolutionarily divergent yeast species following high-temperature stress, and revealed that thermotolerance and s^2^U_34_ level vary markedly among these yeast species. For example, the two commonly used laboratory strains (S288C and W303) of brewer’s yeast *Saccharomyces cerevisiae* show temperature-dependent thiolation deficit that limits its growth to 39 °C^33^. In contrast, in the haploid yeast *Candida glabrata*, which grows well at temperatures up to 43 °C, the thiolation level remains unaltered with the increase of temperature^33^. Furthermore, a single-nucleotide polymorphism in *Ncs2/CTU2* (i.e., H71L) results in a high-temperature growth phenotype^34^, and the thiolation level in *ctu2* mutant of *S. cerevisiae* strain expressing H71L variant of *CTU2* does not exhibit reduced tRNA thiolation at 37 °C compared to that at 30 °C^35^. Therefore, the U_34_ thiolation levels appear to be closely associated with thermotolerance in yeast, and the polymorphism in *CTU2* allele may contribute to different s^2^U_34_ levels and cell growth under elevated temperature. However, it is still unclear whether CTU2 has the conserved function in tRNA thiolation modification in rice. If so, whether the CTU2-mediated tRNA thiolation plays a role in rice response to high-temperature stress is unknown.

Here, we report the identification and characterization of *SLG1* (*Slender Guy 1*) gene, which encodes the cytosolic tRNA 2-thiolation protein 2 (RCTU2) and plays a key role in high-temperature tolerance in rice. We demonstrate that *SLG1* is differentiated between *indica* and *japonica* rice, and the plants with the *indica* allele display enhanced thermotolerance. Our results suggest that artificial selection of *SLG1* during domestication may have contributed to the adaptation of *indica* rice to high-temperature environment.

## Results

### Isolation of the *SLG1* gene

**Fig. 1:**
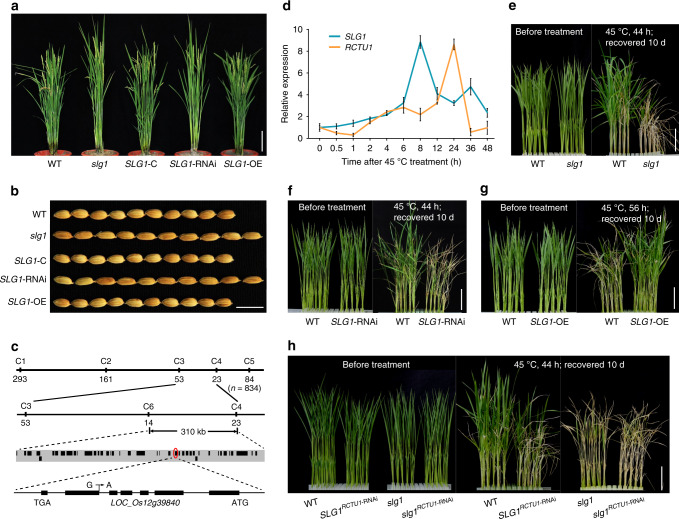
*SLG1* is a positive regulator of thermotolerance. **a**, **b** Phenotypes of whole plant and grain shape of *slg1*, WT, and the transgenic T_2_ lines of *slg1*-C, *SLG1*-RNAi, and *SLG1*-OE. C, complementation; OE, overexpression; RNAi, RNA interference. Bars = 10 cm (**a**) and 1 cm (**b**). **c** Fine mapping of the *SLG1* gene. A single-nucleotide substitution at the splicing acceptor site of the fifth intron of *LOC_Os12g39840* in *slg1* was indicated. **d** *SLG1* and *RCTU1* show high-temperature-inducible expression. Total RNA was extracted from the shoots of 2-leaf-stage seedlings at the indicated time-points. The expression levels were determined by qPCR, and the expression level at time 0 was set to 1. *Actin* was used as the internal control. Data are means ± SD (*n* = 3 biological replicates). **e**–**g** *SLG1* is a positive regulator of thermotolerance. Phenotypes of WT, *slg1*, *SLG1*-RNAi, and *SLG1*-OE plants before and after treatment are shown. Bars = 5 cm. **h** Dysfunction of *RCTU1* shows thermosensitive phenotype similar to the *slg1* mutant. Phenotypes of *RCTU1-*RNAi plants on the background of *slg1* and WT are shown before and after treatment. Bar = 5 cm. The source data underlying (**d**) are provided as a Source data file.

By screening a NaN_3_-mutagenized M_2_ library under the background of KY131 (*temperate japonica*, *Tej*), we identified a mutant with slender plant architecture and increased grain length (Fig. 1a, b and Supplementary Fig. 1a, b, e) and named *slg1* (for *slender guy 1*). Crossing of *slg1* with WT yielded an F_2_ population, in which the segregation ratio of WT and longer grain phenotype was 3:1 (*χ*^2^ = 0.8 < *χ*^2^_0.05_,_1_; 0.5 > *P* > 0.1; null hypothesis: fit 3:1). This result suggests that the *slg1* mutant phenotype is due to the recessive mutation of a single locus. By using 20 bulked WT and mutant plants from the F_2_ population between *slg1* and KD8 (*temperate japonica*), the candidate locus was directly mapped to the long arm of chromosome 12 between markers C1 and C5 (Fig. 1c). Further analysis of 834 F_2_ individuals with mutant phenotype narrowed down the target gene within a 310-kb region between markers C4 and C6 (Fig. 1c). Sequence comparison revealed that there is a single-nucleotide substitution (G to A) at the splicing acceptor site of the fifth intron of *LOC_Os12g39840* in the mutant (Fig. 1c and Supplementary Fig. 2a), resulting in mis-splicing and the formation of multiple transcripts (Supplementary Fig. 2b, c).

To confirm that *LOC_Os12g39840* is indeed *SLG1*, the genomic DNA, including 1637-bp 5′ flanking sequence, *SLG1* coding region, and 840-bp downstream sequence, was amplified from WT and transformed into *slg1*. All of the 21 independent T_2_ transgenic lines (*slg1*-C) showed overall phenotypes resembling that of WT, including grain length and plant architecture (Fig. 1a, b). In addition, transgenic plants with specifically downregulated *SLG1* expression under WT background (*SLG1*-RNAi) exhibited phenotypes similar to that of *slg1* (Fig. 1a, b and Supplementary Fig. 3a), confirming that *LOC_Os12g39840* is indeed the causal gene of the *slg1* mutant.

*SLG1* belongs to the CTU2 superfamily predicted to encode the cytoplasmic tRNA 2-thiolation protein 2 (Supplementary Fig. 4a), and the homologs of SLG1 were widely found in other organisms (Supplementary Fig. 5). Similar to the *Arabidopsis* counterpart of AtCTU2^36^, SLG1 was found to be localized in both nucleus and cytoplasm (Supplementary Fig. 4b), and was ubiquitously expressed (Supplementary Fig. 4c). Moreover, loss-of-function of *SLG1* and *AtCTU2* causes similar defects in root morphology, such as reduced root length and lateral root density, and abnormal development of root hairs (Supplementary Fig. 1f–k)^36^.

### *SLG1* is a positive regulator of thermotolerance

One of the prominent biological functions of CTU1–CTU2 complex in yeast is the response to heat stress^24,32–35^. Expression of *SLG1* was also significantly induced by high temperature (Fig. 1d). *slg1* is highly thermosensitive, ~84% of the mutant seedlings died after being treated at 45 °C for 44 h, while more than 90% of WT survived after recovery (Fig. 1e and Supplementary Fig. 3b). Similarly, the survival rate was also remarkably reduced for the *SLG1*-RNAi plants after high-temperature treatment (Fig. 1f and Supplementary Fig. 3a, c). In contrast, the survival rate of *SLG1*-OE seedlings was much higher (>90%) than that of WT (≈ 60%) under the same stress condition (Fig. 1g and Supplementary Fig. 3a, d), although the overall morphology of the *SLG1*-OE plants, such as grain length and plant architecture, was similar to that of WT under normal conditions (Fig. 1a, b). Taken together, these results indicate that *SLG1* functions positively in rice thermotolerance.

In yeast, CTU2 appears to catalyze the last step of tRNA thiolation process by forming a functional complex with CTU1^23,24,37^. ROL5 is the homologous protein of Ncs6/CTU1 in *Arabidopsis*^38^. Therefore, we used the amino-acid sequence of ROL5/AtCTU1 as the query to search for the homologous proteins in the rice genome. We identified two homologous proteins of LOC_Os02g52470 and LOC_Os01g41565 in rice. LOC_Os02g52470 shares the highest sequence similarity with ROL5/AtCTU1 and is annotated as the rice CTU1 protein (RCTU1). We then conducted the interaction assays to test the presence of CTU1–CTU2 complex both in vivo and in vitro and observed a clear interaction between RCTU1 and SLG1 (Supplementary Fig. 6a–c). To study if RCTU1 works in the same genetic pathway as SLG1, we constructed *RCTU1*-RNAi transgenic plants under the background of WT (*SLG1*^*RCTU1*-RNAi^) and *slg1* (*slg1*^*RCTU1*-RNAi^), respectively. We found that both *SLG1*^*RCTU1*-RNAi^ and *slg1*^*RCTU1*-RNAi^ transgenic plants showed similar phenotypes to *slg1*, including increased grain length, slender plant architecture, and defective root system (Supplementary Figs. 1 and 6d, e). Moreover, similar to *SLG1*, transcription of *RCTU1* was also highly induced by high temperature (Fig. 1d), and the survival rates of *SLG1*^*RCTU1*-RNAi^ and *slg1*^*RCTU1*-RNAi^ seedlings were all greatly reduced under high temperature (Fig. 1h and Supplementary Fig. 3e). These results indicate that the RCTU1-SLG1 complex plays an essential role in rice thermotolerance.

### *SLG1-*mediated thermotolerance is positively correlated with thiolated tRNA levels

**Fig. 2:**
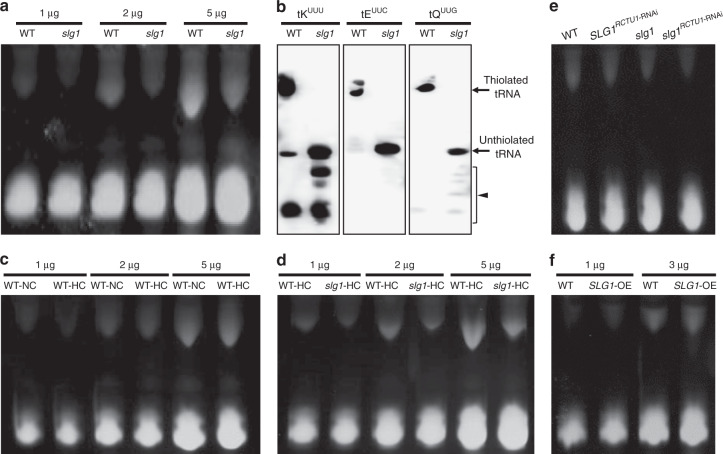
*SLG1*-mediated thermotolerance is positively correlated with thiolated tRNA level. **a** Comparison of thiolated tRNA level between *slg1* and WT. Shoots of 2-leaf-stage seedlings under normal conditions were sampled. The slow-migration band indicates thiolated tRNAs in the presence of APM. The loaded amount of tRNA was 1, 2, and 5 µg for each lane, respectively. **b** Northern blot analysis. The loaded amount of tRNA was 1 µg, which was extracted from 2-leaf-stage normal-grown seedlings. Three tRNA isoacceptors of tK^UUU^, tE^UUC^, and tQ^UUG^ were used to detect the specifically thiolated tRNAs. The bands observed in tK (arrowhead) might be indicative of tRNA halves or tRNA degradation. **c** tRNA thiolation process was adversely affected by high-temperature stress. Shoots of 2-leaf-stage seedlings grown under high-temperature (45 °C, 44 h) and normal conditions were sampled, respectively. The loaded amount of tRNA was 1, 2, and 5 µg for each lane, respectively. NC, normal conditions; HC, high-temperature conditions. **d**–**f** Comparison of thiolated tRNA level. Shoots of 2-leaf-stage seedlings under high-temperature (45 °C, 44 h; **g**) or normal conditions (**h**, **i**) were sampled, respectively. The loaded amount of tRNA was 1, 2, and 5 µg (**d**), 3 µg (**e**), 1 and 3 µg (**f**) for each lane, respectively. The experiments in (**a**–**f**) were repeated three times with similar results. Source data are provided as a Source data file.

It was reported that the CTU1–CTU2 complex is responsible for the 2-thiolation of cytosolic tRNAs, and the loss of thiolation results in the thermosensitive phenotype in the *CTU2-* and *CTU1*-deleted yeast strains^24^. To characterize the role of tRNA thiolation in high-temperature stress response, we first compared the thiolated tRNA levels between *slg1* and WT using a polyacrylamide gel supplemented with APM (N-acryloylamino phenyl mercuric chloride). Thiolated tRNAs can be specifically retarded by APM due to the affinity of the thio group with the mercuric compounds^36^. We found that the amount of thiolated tRNAs in *slg1* was much lower than that in WT (Fig. 2a), suggesting that the loss-of-function of *SLG1* impaired tRNA thiolation in the mutant. The defective tRNA thiolation in *slg1* was further confirmed by Northern blot. We found that dysfunction of *SLG1* specifically impaired the thiolation level of tK^UUU^, tE^UUC^, and tQ^UUG^ (Fig. 2b), which was similarly observed for *Ncs2/CTU2* in yeast. Next, we examined the effect of high temperature on tRNA thiolation by comparing the thiolated tRNA levels under both normal and high-temperature conditions. It was found that elevated temperature influenced the thiolation of U_34_ and caused tRNA hypothiolation in rice (Fig. 2c). The thiolated tRNA levels in WT were much higher than that in *slg1* under high temperature (Fig. 2d), which is consistent with the higher survival rate of WT (Fig. 1e and Supplementary Fig. 3b). With the decreasing level of thiolated tRNAs in the WT, *SLG1*^*RCTU1*-RNAi^, *slg1*, and *slg1*^*RCTU1*-RNAi^ (Fig. 2e), the survival rates of plants were reduced to 90%, 36.5%, 15.2%, and 5%, respectively (Fig. 1h and Supplementary Fig. 3e). In contrast, the *SLG1*-OE plants with increased thiolated tRNA levels conferred enhanced thermotolerance compared with WT (Figs. 1g, 2f, and Supplementary Fig. 3d). Taken together, these results suggest that the *SLG1*-mediated thermotolerance is positively correlated with the thiolated tRNA levels in vivo.

### *SLG1* may have undergone selective sweep in Asian cultivated rice

**Fig. 3:**
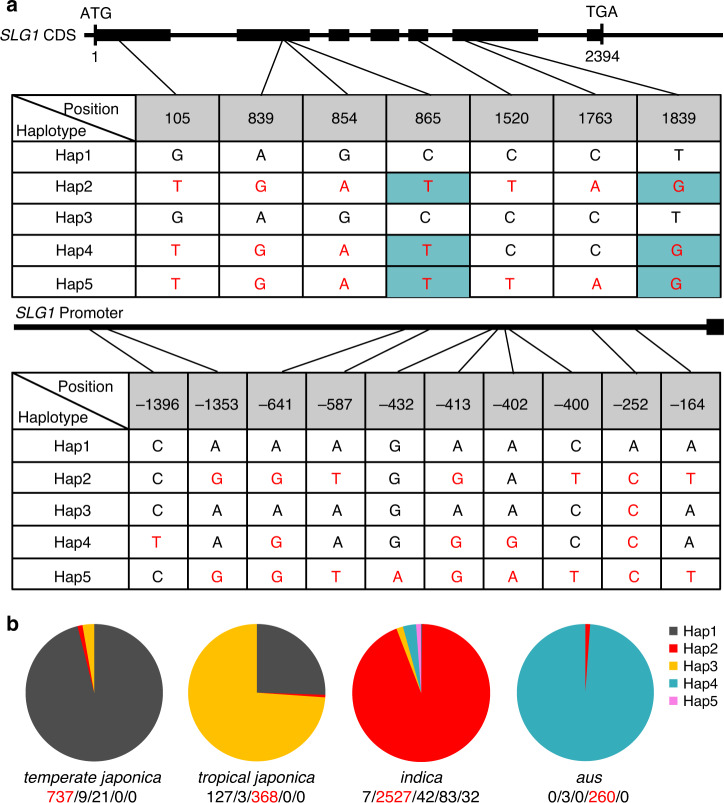
*SLG1* is differentiated in Asian cultivated rice. **a** Haplotype analysis of *SLG1* promoter and coding regions from 4219 Asian cultivated rice accessions. Red letters indicate different nucleotides, and gray-marked letters indicate non-synonymous substitutions. **b** Distribution frequency of the five *SLG1* haplotypes in diverse Asian cultivated rice collection. The number of cultivars for each haplotype was given from left to right below each subpopulation. The haplotype with the largest number was highlighted in red. The source data underlying (**b**) are provided as a Source data file.

In Asian cultivated rice, the two subspecies *indica* and *japonica* are distributed in distinct ecological regions and possess contrasting phenotype under high-temperature stress^39^ (Supplementary Fig. 7a–c). Our results above clearly demonstrated that the thermotolerance of rice is closely related to *SLG1*-mediated tRNA 2-thiolation. A sequence comparison revealed that both the promoter and coding regions of *SLG1* in *japonica* cultivars were different from that in *indica* (*Ind*) cultivars (Supplementary Fig. 8). We further analyzed natural variations of *SLG1* using 4219 sequenced rice accessions^40^, and found ten and seven SNPs in the promoter and coding regions of *SLG1*, respectively (Fig. 3a). Of the seven SNPs in the coding region, the two nucleotide substitutions of T865C and G1839T caused amino-acid conversions from Val^177^ to Ala^177^ and Val^362^ to Phe^362^, respectively, while the other five were synonymous substitutions (Fig. 3a). The two amino-acid mutations were found in cultivated rice, with 98.2% of *indica* containing Val^177^-Val^362^ and 99.1% of *japonica* possessing Ala^177^-Phe^362^, suggesting their contributions to the functional divergence of SLG1.

Based on the polymorphisms, the 4219 accessions could be classified into five haplotypes (Fig. 3b), with 737 out of 767 (96.1%) *temperate japonica* accessions carried Hap1, while 2527 out of 2691 (93.9%) *indica* accessions carried Hap2. Notably, almost all the *aus* accessions (98.9%) could be distinctly classified as Hap4. The *tropical japonica* (*Trj*) group has two major haplotypes, Hap3 (73.9%) and Hap1 (25.5%), which only differ in a single nucleotide (A252C) in the promoter region. Hap5, found only in the *indica* group at a very low frequency (˂0.8%), was highly similar to Hap2 except a single nucleotide difference (G432A) in the promoter region. We further compared the nucleotide sequence of Hap1–Hap4 with 34 *O. rufipogon* wild rice accessions (Supplementary Data 1). Beside the abundant variations across the *SLG1* genomic region among wild rice accessions, a number of *SLG1* haplotypes in wild rice accessions were exactly the same as that of *Ind*, *Tej*, *Trj*, and *Aus*. For example, the *SLG1* allele of the *indica* cultivar 93-11 is identical with that of the wild rice accession W1687 (Hap2); the *aus* cultivar Kasalath shares the same haplotype with W593 (Hap4); the *temperate japonica* cultivar Nipponbare carries the same *SLG1* allele as W3078 (Hap1), while the *tropical japonica* cultivar NERICA3 has the same allele as W120 (Hap3). These results imply that *SLG1*^*Tej*^, *SLG1*^*Trj*^, *SLG1*^*Ind*^, and *SLG1*^*Aus*^ might represent an ancient allele preserved in different *O. rufipogon* wild rice accessions, and might have been subjected to positive selection at early stages of rice domestication.

Regional reduction of nucleotide diversity (π) indicates positive selection^11^. We estimated the nucleotide diversity across the *SLG1* genomic region in the four rice subpopulations. Compared with *O. rufipogon* (π = 0.004098; Supplementary Fig. 9 and Supplementary Table 1), the immediate ancestral progenitor of cultivated rice, the nucleotide diversity of *SLG1* was extremely low in all four cultivated rice subpopulations of *indica* (π = 0.000098), *temperate japonica* (π = 0.000073), *tropical japonica* (π = 0.000541), and *Aus* (π = 0.000073), which was also much lower than that in the whole genome of *O. sativa* (π = 0.0024)^41^. We then measured the ratio of the genetic diversity in wild rice to that in cultivated rice (π_w_/π_c_) across the *SLG1* region, and found the π_w_/π_c_ in all four subpopulations (π_w_/π_c_ = 7–55) was much higher than the top 5% genome-wide threshold used as the selection cutoff (π_w_/π_c_ = 3; Supplementary Table 1)^41^, suggesting that *SLG1* was subjected to strong selection in all these subpopulations. Because positive selection may lead to a selective sweep in the flanking region of the selected gene^11^, we further examined the nucleotide diversity of 30-kb regions flanking *SLG1*. As expected, the average nucleotide diversity of *SLG1* flanking regions in *temperate japonica* (π = 0.000173), *tropical japonica* (π = 0.000542), *indica* (π = 0.000972), and *Aus* (π = 0.000634) were comparable to that of the *SLG1* region but much lower than that in the corresponding wild rice populations (π > 0.0033; Supplementary Table 1). Further, we identified MB-sized low diversity region in both *indica* and *temperate japonica* populations spanning the *SLG1* locus (Supplementary Fig. 9), which is consistent with the selective sweep argument. Regardless, the bottleneck effect may also have its contribution to the diversity signal we have been observing, giving the bottleneck in *japonica* is much intense^41^, which is also revealed by our data (Supplementary Table 1).

To further explore the evolutionary signature of *SLG1* in *indica* and *japonica* subpopulations, we constructed a neighbor-joining tree of *SLG1* and compared to that of random SNPs. Both trees displayed *indica-japonica* segregation and the *SLG1* haplotype of admixture samples also displayed such segregation (Supplementary Fig. 10), suggesting the lack of introgression in *SLG1*. Further, we calculated the fixation index (*F*_ST_) around *SLG1* among different populations, and observed a high level of differentiation between the *indica* and *temperate japonica* populations, confirming the lack of introgression between the two populations (Supplementary Fig. 11). To be noted, the selective sweep region (Supplementary Fig. 9) contains over 500 genes including *SLG1*, any of which could be the driving factor of the selective sweep. Nevertheless, these data suggested that the differentiation of *SLG1* was driven by selection acting on this region.

### *SLG1*^*Ind*^-carrying plants show strong thermotolerance

The USDA Mini-Core germplasms are a diverse collection of landraces that were not derived from modern breeding^42^. Through mapping the geological distribution of *SLG1* haplotypes in Mini-Core germplasms alongside with the 30-year (1960–1990) average terrestrial temperature, we found that Hap2, the allele carried by 93.9% *indica* accessions, is predominantly distributed in the regions with annual mean temperature above 29 °C. In contrast, Hap1, the allele carried by 96.1% *temperate japonica* accessions, is predominant in the regions with annual mean temperature below 18 °C (Supplementary Fig. 12a). And the similar geographic distribution was observed when plotting the *SLG1*-Hap1/Hap2 distribution in admixture germplasms alongside with the average terrestrial temperature (Supplementary Fig. 12b). These results suggest that the Hap1 and Hap2 haplotypes of *SLG1* might have potential contribution for different environmental-temperature adaptation of rice.

**Fig. 4:**
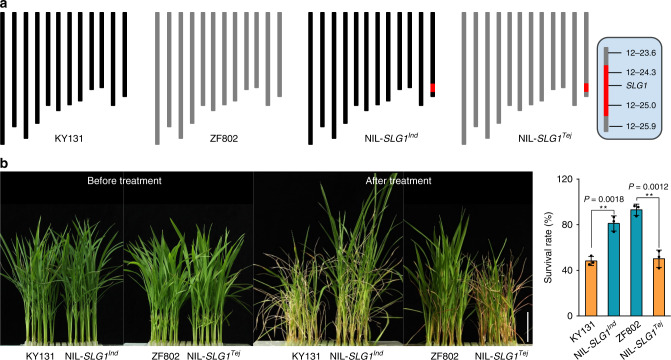
*SLG1*^*Ind*^ confers higher thermotolerance than *SLG1*^*Tej*^. **a** Chromosome maps of NIL-*SLG1*^*Ind*^ and NIL-*SLG1*^*Tej*^. Red rectangles indicate the donor segment containing the *SLG1* locus. NIL-*SLG1*^*Ind*^ indicates KY131 (*temperate japonica*) with *SLG1*^*Ind*^ allele from ZF802 (*indica*), and NIL-*SLG1*^*Tej*^ indicates ZF802 with *SLG1*^*Tej*^ allele from KY131. **b** Comparison of thermotolerance between *SLG1*^*Ind*^ and *SLG1*^*Tej*^ alleles. Two-leaf-stage seedlings were treated with 45 °C for 56 h for KY131 and NIL-*SLG1*^*Ind*^, and 64 h for ZF802 and NIL-*SLG1*^*Tej*^, respectively. The seedlings were then recovered under normal conditions for 10 days. Data are means ± SD (*n* = 3 biological replicates, with 80–96 seedlings per biological replicate). **P < 0.01, two-tailed Student’s *t*-test. Bar = 5 cm. The source data underlying (**b**) are provided as a Source data file.

In view of the fact that *indica* and *japonica* rice have experienced clear adaptation to distinct environmental temperature^9,39,41,43^, and the important role of *SLG1* in thermotolerance, we speculate that the differentiation of *SLG1* between *indica* and *japonica* might be one of the decisive factors for the two subspecies to tolerate different environmental temperature. Therefore, we constructed near-isogenic lines (NIL) by introducing the *indica* allele of *SLG1* (*SLG1*^*Ind*^) into the background of KY131 (*SLG1*^*Tej*^), and *vice versa* (Fig. 4a). The obtained NIL-*SLG1*^*Ind*^ and NIL-*SLG1*^*Tej*^ plants exhibited the same appearance as their corresponding recipients at seedling stage under normal growth conditions (Fig. 4b). When encountering high-temperature stress, the survival rate of NIL-*SLG1*^*Tej*^ seedlings was much lower (50%) than that of the *indica* recipient ZF802 (93%) (Fig. 4b). In contrast, the NIL-*SLG1*^*Ind*^ seedlings exhibited markedly enhanced thermotolerance relative to KY131, with the mean survival rate of about 81% in NIL-*SLG1*^*Ind*^ compared with that of about 48% in KY131 (Fig. 4b), indicating that *SLG1*^*Ind*^ is superior to *SLG1*^*Tej*^ in plant tolerance to high-temperature stress.

**Fig. 5:**
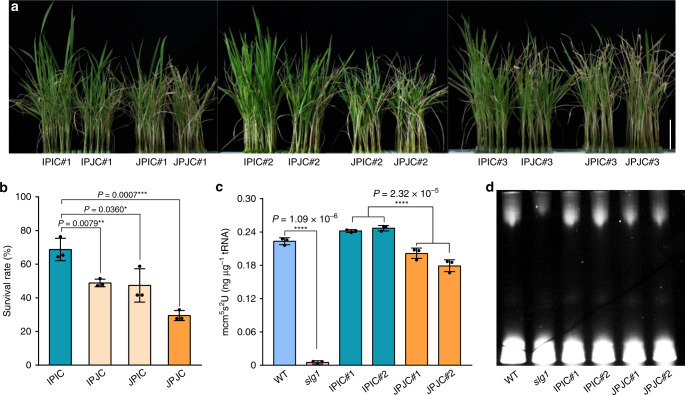
*SLG1*^*Ind*^-complemented plants show strong thermotolerance and high thiolated tRNA level. **a**, **b** Comparison of thermotolerance among IPIC, IPJC, JPIC, and JPJC plants. Three independent homozygous IPIC, IPJC, JPIC, and JPJC lines were selected and treated with 45 °C for 56 h and then recovered under normal condition for 10 days. Data are means ± SD (*n* = 3 independent transgenic lines. Each transgenic line was analyzed with three biological replicates, with 40–56 seedlings per biological replicate). *P < 0.05, **P < 0.01, ***P < 0.001, two-tailed Student’s *t*-test. Bar = 5 cm. **c**, **d** Detection of thiolated tRNAs in *slg1*, WT, IPIC, and JPJC transgenic lines based on LC/MS-QQQ- (**c**) and APM- (**d**) methods. Shoots of 2-leaf-stage seedlings under normal growth conditions were sampled. The loaded amount of tRNA was 3 µg for each lane (**d**). Data are means ± SD (*n* = 3 biological replicates). ****P < 0.0001, two-tailed Student’s *t*-test. The experiment in (**d**) was repeated three times with similar results. The source data underlying (**b**–**d**) are provided as a Source data file.

To further evaluate the functional difference between *SLG1*^*Ind*^ and *SLG1*^*Tej*^, we adopted transgenic approach by amplifying the promoter and gene sequences of *SLG1* from KY131 and 93-11, and introduced them into *slg1* to generate complementary plants JPJC (the *Tej*-type promoter with *Tej*-type gene sequences) and IPIC (the *Ind*-type promoter with *Ind*-type gene sequences), respectively (Supplementary Fig. 13a). We planted 32 plants for each transgenic line and observed overall agronomic traits including plant height, heading date, grain length, grain width, and seed-setting rate under normal growth conditions in three growing seasons, and three independent T_3_ transgenic lines with WT-phenotype were selected for thermotolerance comparison. We found that the IPIC lines exhibited much higher thermotolerance than that of JPJC, with the mean survival rate of about 69% in IPIC compared with that of about 29% in JPJC (Fig. 5a, b). The enhanced thermotolerance conferred by *SLG1*^*Ind*^ was in accordance with the better adaptability of *indica* to the tropical or subtropical regions. Notably, the overall gross morphology of the IPIC plants including grain length and plant architecture was quite similar to that of WT and JPJC plants under normal growth conditions (Supplementary Figs. 13b, c and 14), suggesting that the *SLG1*^*Ind*^ allele has great potential in thermotolerance breeding.

To understand the role of tRNA thiolation in differential thermotolerance of *SLG1*^*Ind*^ and *SLG1*^*Tej*^, we quantified the thiolated tRNAs in IPIC and JPJC plants by liquid chromatography-tandem quadrupole mass spectrometry (LC/MS-QQQ) using synthetic mcm^5^s^2^U as the standard. Similar to the results of APM-PAGE analysis (Fig. 2a), the thiolated tRNA level in *slg1* was markedly decreased compared to that in WT (Fig. 5c), and introduction of either *SLG1*^*Ind*^ or *SLG1*^*Tej*^ rescued the thiolation defect of the mutant (Fig. 5c). Notably, we found that the mcm^5^s^2^U amounts in IPIC were much higher than that in JPJC (Fig. 5c), which was also observed by the APM-PAGE method (Fig. 5d). Taken together, these results strongly suggest that *SLG1*-mediated tRNA thiolation is responsible for the difference in thermotolerance between *SLG1*^*Ind*^ and *SLG1*^*Tej*^.

### The variations at both promoter and coding sequences of *SLG1* contribute to thermotolerance

In addition to the ten SNPs found in the 1637-bp promoter region of *SLG1*, seven InDels were also found in this region (Supplementary Fig. 8). It was unclear whether the polymorphism of the promoter region or the conversion of two amino acids in coding region was responsible for the differential thermotolerance between *SLG1*^*Tej*^ and *SLG1*^*Ind*^. Although the 1637-bp promoter of *SLG1* was sufficient for complementary test (Supplementary Fig. 13), the luciferase activity of *pSLG1*-*LUC* was extremely low in both constructs of *SLG1*^*Tej*^ and *SLG1*^*Ind*^ in vitro (Supplementary Fig. 15a). We speculate that other regulatory elements may need for *SLG1* transcription. Therefore, the activity of the two promoters of *SLG1*^*Ind*^ and *SLG1*^*Tej*^ in protoplasts may not reflect the situation in vivo. In contrast, the in vivo transcription of *SLG1* was significantly higher in *indica* cultivars even under normal growth conditions (*P* = 0.0016, two-tailed *t*-test; Supplementary Fig. 7d), and higher level of *SLG1* expression in NIL-*SLG1*^*Ind*^ (KY131 background) and IPIC plants was also observed under either normal condition or heat stress compared with that in the recipient KY131 and JPJC plants, respectively (Supplementary Fig. 15b–d). Together with the thermosensitivity of *SLG1*-RNAi plants and the thermotolerance of *SLG1*-OE plants (Fig. 1f, g), these results clearly demonstrate the positive correlation between *SLG1* transcription level and thermotolerance.

To explore whether the polymorphism of promoter regions results in the difference of *SLG1* expression, we developed two promoter-swapping vectors JPIC (the *Tej*-type promoter with *Ind*-type gene sequences) and IPJC (the *Ind*-type promoter with *Tej*-type gene sequences) using the 1637-bp promoter and gene sequences of *SLG1*, and were then transformed into *slg1* mutant, respectively (Supplementary Fig. 13a). Under normal growth conditions, the gross morphology and grain shape among IPIC, IPJC, JPIC, and JPJC plants were comparable to each other (Supplementary Fig. 13b, c). When subjecting the four complementary seedlings to high-temperature stress, we found that the survival rate of IPIC plants was the highest (69%), while that of JPJC plants was the lowest (29%). Interestingly, the survival rates of IPJC plants (49%) and JPIC plants (47%) were significantly higher than that of JPJC plants but lower than that of IPIC plants (*P* < 0.05, two-tailed *t*-test), while no significant difference was observed between IPJC plants and JPIC plants (*P* = 0.8160, two-tailed *t*-test) (Fig. 5a, b). These results indicate that the variations at both promoter and coding regions collectively contribute to the *SLG1*-mediated thermotolerance.

### *SLG1*^*Ind*^ enhances thermotolerance at the reproductive stage

**Fig. 6:**
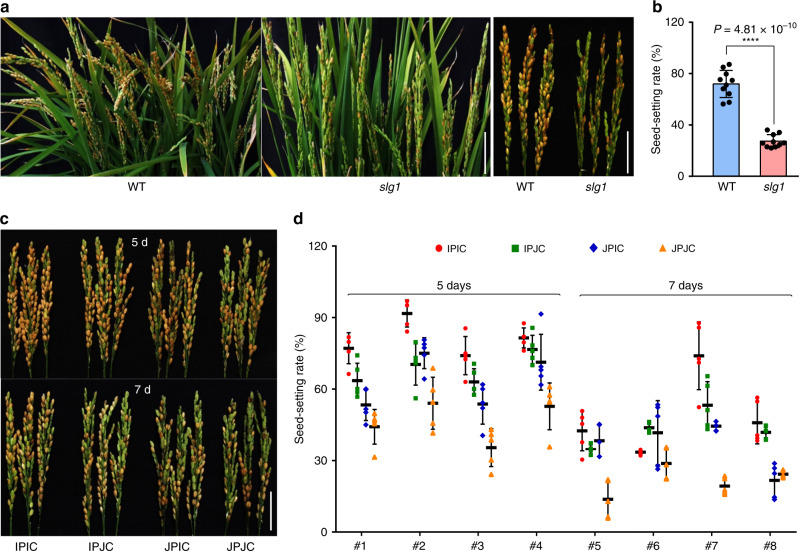
*SLG1*^*Ind*^ enhances thermotolerance at the reproductive stage. **a**, **b** The *slg1* mutant is highly sensitive to high temperature at the reproductive stage. Seed-setting rate was determined after treatment under 14 h light (40 °C)/10 h dark (31 °C) for 5 days, and recovered under normal conditions until seed maturation. Data are means ± SD (*n* = 10 panicles with three biological replicates). ****P < 0.0001, two-tailed Student’s *t*-test. Bar = 5 cm. **c**, **d** Comparison of thermotolerance among IPIC, IPJC, JPIC, and JPJC plants. Eight independent homozygous IPIC, IPJC, JPIC, and JPJC lines were treated with 14 h light (40 °C)/10 h dark (31 °C) for 5 days and 7 days, respectively, and then recovered under normal conditions until seed maturation. The *x*-axis indicates different transgenic lines. Data are means ± SD (*n* = 5 panicles with three biological replicates). Bar = 5 cm. The source data underlying (**b**), (**d**) are provided as a Source data file.

Rice plant is highly sensitive to high-temperature stress especially at the reproductive stage^44^, and improving thermotolerance at the reproductive stage is thus of particular importance to reduce yield loss. Therefore, we performed high-temperature treatment of the adult plants of *slg1* and WT to evaluate the role of *SLG1* at the reproductive stage. We found that the seed-setting rate of *slg1* was less than 28%, while that of WT could reach to 72% after treatment at 40 °C for five days (Fig. 6a, b), suggesting that the *slg1* mutant was also sensitive to high-temperature stress at the reproductive stage. Microscopic examination found that the pollen viability of *slg1* was moderately reduced compared with that of WT under the stress condition (Supplementary Fig. 16a), but the number of pollen grains on the stigma of *slg1* was significantly reduced (Supplementary Fig. 16b, c), and pollen-tube elongation could be hardly observed in the *slg1* mutant under high-temperature treatment (Supplementary Fig. 16d). These results demonstrated that *SLG1* plays an important role in thermotolerance at the reproductive stage.

In our NIL lines, we observed a decreased fertility for NIL-*SLG1*^*Ind*^ plants (Supplementary Fig. 17), which is likely due to the tight linkage of *SLG1* with the hybrid sterility gene *HSA1*^45^. To further explore the possible role of *SLG1* at the reproductive stage, we selected eight independent lines from each of the IPIC, IPJC, JPIC, and JPJC constructs, half of which were treated with high temperature for 5 days and the other half for 7 days at the microsporocyte meiosis stage, respectively. Among the four complementary lines, the IPIC lines showed the strongest thermotolerance in terms of seed-setting rate, which is about 81% and 49% after 5 and 7 days high-temperature treatment, respectively (Fig. 6c, d and Supplementary Fig. 18). In contrast, the seed-setting rate of JPJC was only about 47% and 22%, respectively, under the same stress condition (Fig. 6c, d and Supplementary Fig. 18). Again, we could not distinguish the function of the promoter from that of the coding sequence, because there was no significant difference in the seed-setting rate between IPJC and JPIC lines (*P* > 0.30, two-tailed *t*-test). The seed-setting rates of IPJC lines after 5 and 7 days of treatment were about 68% and 43%, respectively, while those of JPIC lines were about 63% and 37% (Fig. 6c, d and Supplementary Fig. 18). These results further suggest that variations at both promoter and coding sequences of *SLG1*^*Ind*^ contribute to the thermotolerance in rice.

## Discussion

With global warming and climate change, understanding how plants respond to high-temperature stress and breeding thermotolerant crops are urgently needed for sustainable agriculture. However, few thermotolerance-related genes have so far been identified and characterized. In this study, we provided several lines of evidence that tRNA thiolation plays an important role in rice thermotolerance. First, *SLG1* and *RCTU1*, the rice homologous genes of yeast *Ncs2/CTU2* and *Ncs6/CTU1*, respectively, were strongly induced by high temperature (Fig. 1d). Second, dysfunction of SLG1 resulted in a significant decrease in the thiolated tRNA level in vivo (Figs. 2a, b and 5c), and the corresponding mutant displayed thermosensitive phenotype (Fig. 1e and Supplementary Fig. 3b). Third, the survival rates of WT, *SLG1*^*RCTU1*-RNAi^, *slg1*, and *slg1*^*RCTU1*-RNAi^ decreased after high-temperature treatment (Fig. 1h and Supplementary Fig. 3e), which were consistent well with the decreasing level of thiolated tRNAs in these plants (Fig. 2e). Fourth, overexpression of *SLG1* increased the thiolated tRNA level and enhanced the thermotolerance of the transgenic plants (Figs. 1g, 2f, and Supplementary Fig. 3d). Fifth, the thiolated tRNA level of the complementary IPIC plants was higher than that of the JPJC plants (Fig. 5c, d), and the thermotolerance of the former was also much better than that of the latter (Figs. 5a, b and 6c, d). In addition, the complementary lines of IPIC and JPJC displayed differences in thiolated tRNA level and thermotolerance (Figs. 5 and 6c, d), although their morphological traits were similar to WT under normal growth conditions (Supplementary Figs. 13 and 14). Therefore, our results revealed the importance of *SLG1*-mediated tRNA thiolation in rice thermotolerance.

Numerous studies have revealed that tRNA thiolation of U_34_ plays a critical role in fine-tuning protein translation. Lacking of U_34_ thiolation in tK^UUU^, tE^UUC^, and tQ^UUG^ leads to ribosome pausing at their cognate codons, thus influencing the rate of translation and protein folding, and causing proteotoxic stress and endogenous protein aggregation in yeast^27,46,47^. Further, we explored the effects of tRNA thiolation on proteome, and found that dysfunction of *SLG1* affects the accumulation of plenty of proteins, especially those involved in translation, metabolism, and stress responses (Supplementary Data 2 and Supplementary Fig. 19). The extensive influence of tRNA thiolation defects on protein homeostasis would trigger chronic proteotoxic stress^27^. Previous studies have shown that protein-synthesis defects in the URM1 pathway mutants in yeast led to the production of misfolded or error-containing proteins^27,46^, which triggered endoplasmic reticulum stress, thus activating downstream signaling pathways involved in abiotic-stress responses^46,48^. Similarly, our proteomic analysis also revealed the accumulation of five proteins (TrEMBL UniProt ID: Q6ZFJ9, Q651B0, Q653F6, Q6Z2M2, and Q9LWT6) in the *slg1* mutant (Supplementary Data 2), these proteins appear to be involved in unfolded protein binding and chaperone-mediated protein folding. Moreover, we found that some oxidative stress-related proteins, such as TrEMBL UniProt ID Q0E4K1, Q0DTX5, P93407, and Q6L4W7 (Supplementary Data 2), were abnormally synthesized in the mutant, which might be due to the interplay between unfolded protein response and reactive oxygen species (ROS) signaling^49^. Therefore, the tRNA thiolation status *in planta* appears to be closely related to ROS homeostasis^38^, which might be beneficial for the *SLG1*^*Ind*^-carrying plants to tolerate high-temperature stress.

**Fig. 7:**
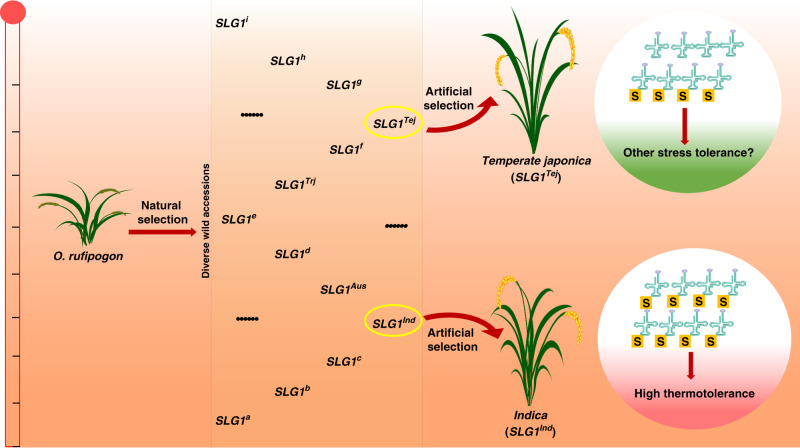
Selection model of the *SLG1* alleles during domestication. *SLG1* is diversified among wild rice accessions. The intimate association of tRNA thiolation and thermotolerance led to the artificial selection of the *SLG1*^*Ind*^ allele for the adaptation of *indica* rice to geographical regions with high environmental temperature. Interestingly, the *SLG1*^*Tej*^ allele was also subjected to selection during *japonica* domestication, which might be due to the dynamic response of tRNA thiolation to various environmental stresses^46^.

The two Asian cultivated subspecies, *indica* and *japonica*, are distributed worldwide from tropical to temperate regions. Typical *indica* and *japonica* cultivars have experienced local adaptation to different environmental niches during the long-term natural and artificial selection^9^. Recent works demonstrate that several genes such as *COLD1*, *CTB4a*, and *bZIP73*, contribute to the cold adaptation during *japonica* domestication^10,11,13^. Here, we showed that the *SLG1* gene is differentiated between *indica* and *japonica*, and plants carrying *SLG1*^*Ind*^ were more thermotolerant than those harboring *SLG1*^*Tej*^ at both seedling and reproductive stages, which was consistent with the distinct geographical distribution of the two subspecies. The sharply reduced nucleotide diversity of *SLG1* strongly suggests that it may have been subjected to positive selection during domestication (Supplementary Fig. 9 and Supplementary Table 1). However, unlike other genes identified with specific functional nucleotide polymorphisms selected during domestication^8,10,11,13^, *SLG1* showed variations in both promoter and coding sequences which collectively contribute to thermotolerance. Although the variations across *SLG1* genomic region among wild rice accessions were highly abundant, most of the *indica* accessions carried Hap2 and were distributed predominantly in geographical regions with higher environmental temperature, suggesting that the domestication of *indica* rice was associated with strong selection of favored *SLG1* alleles that enhanced thermotolerance for growth in regions with higher temperature (Fig. 7).

In summary, we demonstrate the role of tRNA thiolation in rice thermotolerance. Our results suggest that tRNA thiolation pathway might be a suitable target for breeding rice varieties with better tolerance to high-temperature stress. The greater diversity of *SLG1* sequence in *O. rufipogon* wild rice accessions, combining with genome editing technique, may help us to select or generate excellent alleles with higher thermotolerance, which may contribute to the next generation rice breeding for the changing climate and warming globe.

## Methods

### Plant materials and growth conditions

The *slg1* mutant was identified from the sodium azide mutagenized M_2_ population under the background of KY131, and the mapping population was derived from the cross of *slg1* with KD8. For NIL construction, reciprocal cross was performed between ZF802 and KY131, and the resultant F_1_ plants were backcrossed with ZF802 and KY131, respectively. The backcrossed progeny was genotyped with 111 polymorphic markers evenly distributed across the whole rice genome (Supplementary Fig. 20), and NIL-*SLG1*^*Tej*^ and NIL-*SLG1*^*Ind*^ were selected in the BC_5_F_2_ generation. After two more rounds of self-fertilization, the homozygous BC_5_F_4_ populations were used for phenotyping. For IPIC, IPJC, JPIC, and JPJC transgenic lines, 32 plants for each line were planted, and overall agronomic traits, including plant height, heading date, grain length, grain width, and seed-setting rate, were observed under normal growth conditions in three growing seasons, and the lines with WT-phenotype were selected for expression analysis and thermotolerance comparison. All the materials used in this study were cultivated in the experimental fields of the Institute of Genetics and Developmental Biology in Changping (40.2°N, 116.2°E), Beijing during summer, and Lingshui (18.5°N, 110.0°E), Hainan province during winter, respectively.

### Thermotolerance assay

For high-temperature treatment at the seedling stage, healthy seeds were surface-sterilized with 3% sodium hypochlorite for 30 min, soaked at 37 °C for 3 days. Germinating seeds were sowed into 96-well plates, and water-cultured in the phytotron (SANYO) with 14 h light (28 °C)/10 h dark (25 °C), 65–70% relative humidity, and 150 µM m^−2^ s^−1^ photon flux density. Five-day-old pre-cultured seedlings were then transferred to outdoor natural conditions for further growth. Seedlings of 2-leaf stage were treated with 45 °C ± 1 °C for a given time in the above-described phytotron (SANYO), and then recovered under normal conditions for 10 days. The survival rate was calculated as the ratio of the number of seedlings with new green leaves to the total number of treated seedlings.

For high-temperature treatment at the reproductive stage, germinating seeds were sowed on April 16, April 26, and May 12, respectively, and the seedlings of 4-leaf stage were transplanted into pots (25 cm × 25 cm × 35 cm) filled with the same amount of paddy soil, each pot with four plants, and cultivated under natural growth conditions. The tillers at the microsporocyte meiosis stage were tagged and transferred into the phytotron (PERCIVAL, USA), with the photon flux density of 500 µmol m^−2^ s^−1^ and relative humidity of 75–80%. High-temperature treatment was performed under 40 °C during the day (06:00–20:00) and 31 °C at night (20:00–06:00) for 5 and 7 days, respectively, and the plants were then recovered under normal growth conditions until seed maturation. Seed-setting rate was calculated as the percentage of the number of filled grains to the number of total spikelets in a panicle.

### Map-base cloning

The mapping population was derived from the cross of *slg1* with KD8, and whole-genome polymorphic markers were designed based on resequencing data (30×). Using 20 bulked WT and mutant plants, respectively, the candidate gene was first mapped to the long arm of chromosome 12 between markers C1 and C5. Further analysis of the F_2_ mutant plants subsequently fine-mapped the casual gene to the region between C4 and C6. Sequence comparisons between *slg1* and WT were then performed for all the annotated genes within this region, and a single-nucleotide substitution (G to A) at the splicing acceptor site of the fifth intron of *LOC_Os12g39840* was identified in the mutant. This mutation led to the formation of at least four kinds of splicing variants of *SLG1* in the mutant, which were confirmed by sequencing, and no normal *SLG1* transcript was found in the mutant. The primer set used for amplifying the splicing variants is listed in Supplementary Data 3.

### Plasmid construction and plant transformation

For JPJC and IPIC complementary plasmids, a *SLG1* genomic DNA containing the 1637 bp fragment upstream of ATG, coding region, and 840 bp downstream sequence was amplified from KY131 (*SLG1*^*Tej*^) and 93-11 (*SLG1*^*Ind*^), respectively, and then ligated into the binary vector pZH2B using *Xba*I and *Sac*I. For JPIC and IPJC constructs, *Xba*I and *Apa*I were first used to cut the JPJC and IPIC complementary plasmids, respectively, to obtain the promoter sequence of *SLG1*^*Tej*^ and *SLG1*^*Ind*^ alleles. Next, *Apa*I and *Sac*I were used to cut the JPJC and IPIC plasmids, respectively, to obtain the rest of the DNA fragment. Then, the *SLG1*^*Tej*^ promoter and the rest of *SLG1*^*Ind*^ DNA fragment were integrated with T_4_ ligase to produce the JPIC construct, and the *SLG1*^*Ind*^ promoter and the rest of *SLG1*^*Tej*^ DNA fragment were ligated to generate IPJC construct (Supplementary Fig. 13a). For *SLG1*-RNAi and *RCTU1*-RNAi plasmids, the hairpin sequence with two ~300 bp cDNA inverted repeats was inserted into the binary vector pZH2Bi, and introduced into *slg1* and KY131 background, respectively. For overexpression construct, the full-length coding sequence of *SLG1* was amplified from KY131 and ligated into pZH2Bi driven by the ubiquitin promoter, and introduced into the KY131 background. For Flag-tagged *SLG1* overexpressing plasmid, the full-length coding sequence of *SLG1* was amplified from KY131 and ligated into Cambia 1300-221-Flag vector driven by the 35S promoter, then introduced into the *slg1* mutant background. Primers used for vector construction are listed in Supplementary Data 3. Rice transformation was carried out by *Agrobacterium*-mediated method.

### Quantitative real-time RT-PCR

Total RNA was isolated from different tissues using the RNAiso Plus Kit (TaKaRa). About 1 µg RNA was treated with DNase I (Promega) and reversely transcribed using a reverse transcription kit (Promega). The qRT-PCR assay was carried out using SYBR Green I Master reagent and a Light Cycler Nano system (Roche). The *Actin* gene was used as the internal control for normalization. Primers used for expression analysis are listed in Supplementary Data 3.

### Microscopic examination

For root hair examination, healthy seeds were sterilized by 2% sodium hypochlorite for 30 min, and then sowed in 1/2 MS solid medium for 3 days under the photoperiod of 14 h light (28 °C)/10 h dark (25 °C). The seminal roots of about 1 cm in length were cut off and immersed in the liquid nitrogen, and root hair morphology was observed under a cryo-scanning electron microscope (S-3000N, Hitachi).

Anthers and florets were collected from the plants grown under normal and high-temperature (40 °C, 5 days) conditions, respectively, at the flowering stage. The anthers were then stained with 1% I_2_-KI solution to evaluate pollen fertility, and the florets were stained with 0.5% aniline blue solution for 12 h to observe the amount of pollens on the stigmas. To visualize pollen-tube growth, the pistils from the plants ~30 min after flowering grown under normal and high-temperature conditions were collected, and fixed in the solution containing 30% chloroform, 10% acetic acid, and 57% ethanol. After washing with deionized water, pistils were incubated in 10 mol/L NaOH under 56 °C for 8 min and stained with 0.1% aniline blue solution for 12 h. The above samples were then photographed under the wavelength of 350 nm using a microscope (BX53, Olympus).

### tRNA extraction and quantification

Shoots of 50 seedlings at 2-leaf-stage grown under normal and high-temperature (45 °C, 44 h) conditions, respectively, were collected for each extraction. tRNA was extracted according to the previously described method with some modifications^36^. Briefly, samples were ground in liquid nitrogen and suspended in 6 mL 0.9% NaCl, and the suspension was transferred to an RNAase-free 50-mL falcon tube. After adding 12 mL acid phenol (Sigma) and 1.2 mL chloroform, and mixing in a vortex for 30 s, the mixture was centrifuged at 8600 *×* *g* for 10 min. Then, the supernatant was transferred to a new 50-mL falcon tube with the addition of 6 mL acidic phenol and 0.6 mL chloroform. Repeat the above steps for 2–3 times until the interphase was moderately clean. After equilibrating with 10 mL buffer [10 mM Tris (pH 6.3), 15% ethanol, 200 mM KCl, 0.15% Triton X-100], the Nucleobond AX100 columns (Macherey Nagel) were then used to bind the tRNA in the above supernatant. After washing twice with 12 mL buffer [10 mM Tris (pH 6.3), 15% ethanol, 300 mM KCl], the columns were then eluted with 10 mL elution buffer [10 mM Tris (pH 6.3), 15% ethanol, 650 mM KCl], and the effluent was collected into a new falcon tube containing 25 mL absolute ethanol and gently mixed. After standing overnight at −20 °C, the mixture was centrifuged at 8600 *×* *g* for 10 min at room temperature. The precipitate was then washed 2–3 times with 80% ethanol, dried at room temperature, and dissolved with 40 μL DEPC H_2_O.

For APM-PAGE, the above extracted tRNA was denatured at 80 °C for 5 min and separated on a 10% acrylamide gel supplemented with 50 µg/mL APM (TRC, Canada). The resulting gel was stained with ethidium bromide and photographed using the Bio Doc-It^TM^ imaging system.

For Northern blots, tRNAs from WT and *slg1* were separated by APM-PAGE gels as described above, and the gel was transferred to positively charged Nylon membrane (Roche) with Semi-dry Transfer System (BIO-RAD, 170-3940). Then the membrane was blotted following the instructions for DIG Northern Starter Kit (Roche). The DNA probes were synthesized following the sequences listed in Supplementary Data 3 and DIG-labeled (Roche).

For LC/MS-QQQ analysis, about 40 µg tRNA from different samples was digested with Nuclease P1 (Sigma: N8630) and bacterial alkaline phosphatase (Sigma: P4252), and the hydrolysate diluted with an equal volume of water was then analyzed by LC/MS-QQQ. Calibration curve of mcm^5^s^2^U was obtained with different concentrations of authentic standard. The amount of mcm^5^s^2^U in standard samples was determined by the linear regression of the peak area vs its concentration. And the mcm^5^s^2^U concentration in individual samples was quantified with the calibration curve. Chromatographic separation was performed on a ZORBAX Elipse Plus C18 RRHD column (100 mm × 2.1 mm ID, 1.8 µm, Agilent). The amount of tRNA sample loaded onto the column was 1 µL (0.286 µg/µL). Mobile phase was as following: solution A (10% acetonitrile, 5 mM ammonium formate); solution B (90% acetonitrile, 5 mM ammonium formate). Flow rate was 0.35 mL/min. Gradient condition of the mobile phase was as follows: 0–0.5 min, 100% solution A; 0.5–3.0 min, 100% B; 3.0–4.0 min, 100% buffer B. Data were acquired by 1290 UPLC/G6495A QQQ MS (Agilent, USA) in MRM mode. The transitions from precursor ions to product ion are *m/z* 333 to 201 (CE = 10 v) in positive mode for mcm^5^s^2^U. The parameters of MS detection were as following: source temperature is 100 °C; gas temperature is 200 °C; gas flow is 15 L/min; nebulizer is 30 psi; sheath gas heater temperature is 350 °C; sheath gas flow is 12 L/min. The data are processed by the Masshunter software.

### Transient expression in rice protoplasts

For subcellular localization, the full-length coding sequences of *SLG1* and *AtCTU2* were cloned into pSAT6-EYFP-N1 vector to form *SLG1*-*EYFP* and *AtCTU2*-*EYFP* constructs, respectively, and empty vector was used as a negative control. OsbZIP52-RFP was used as the nuclear localization marker. For BiFC assay, the full-length coding sequences of *SLG1* and *RCTU1* were cloned into pUC19-VYNE(R) and pUC19-VYCE(R) vectors and fused with either N- or C-terminal of Venus sequence, respectively. These constructs and corresponding empty vectors were transformed into rice protoplasts. After incubation in dark for 16 h, YFP, RFP, and Venus signals were observed using laser scanning confocal microscope (Leica TCS SP5).

For promoter activity assay, the *SLG1* promoter sequences used for the complementary test were amplified from Nipponbare (*pSLG1*^*Tej*^) and 93-11 (*pSLG1*^*Ind*^), respectively, and cloned into pGreenII 0800-LUC vector containing the firefly luciferase (fLUC) gene and the *Renilla* LUC gene (rLUC). These constructs and corresponding empty vectors were transformed into rice protoplasts, respectively. The promoter of *Chalk5* was used as a positive control^50^. After incubation in dark for 16 h, protoplasts were collected and luciferase activity was measured as described in the Dual-Luciferase® Reporter Assay System Manual (Promega).

### Yeast two-hybrid assay

The full-length coding sequences of *SLG1* and *RCTU1* were amplified from KY131 and cloned into pGBKT7 or pGADT7 vector (Clontech), respectively. The resulting constructs and the corresponding empty vectors were transformed into Golden Yeast according to the Clontech Yeast Two-Hybrid System User Manual, and interactions were detected on SD/-Leu-Trp-His-Ade medium.

### Co-immunoprecipitation

Rice protoplasts from Flag-tagged transgenic plants overexpressing *SLG1* were transformed with plasmid containing 6× Myc-tagged RCTU1 expression cassette. After 16 h incubation, the protoplasts were collected by centrifugation at 150 *×* *g* and the pellet was resuspended with protein extraction buffer [50 mM Tris-HCl (pH 7.5), 150 mM NaCl, 5 mM EDTA, 0.1% Triton X-100, 0.2% Nonidet P 40, 0.6 mM PMSF, and 1× Protein Inhibitor Cocktail (Roche)]. After 30 min gentle rotation at 4 °C, the lysate was centrifuged at 4 °C with 14,000 *×* *g*, and the supernatant was co-incubated for 16 h with anti-Flag beads (Merck, M8823-1ML) prewashed with protein extraction buffer. The beads were then washed five times with protein extraction buffer and boiled in 1× SDS sample buffer for 5 min. The supernatant was used for western blotting analysis. Anti-flag (Sigma, F1804) and anti-Myc (Abcam, ab9106) were used for the detection of immunoprecipitated flag-tagged and Myc-tagged protein in the western blot analysis, respectively. The antibodies were all diluted by 1000 times.

### Haplotype analysis

The 1637-bp promoter and full-length coding sequence of *SLG1* in 4219 cultivated rice accessions were retrieved from the rice VarMap2 database (http://ricevarmap.ncpgr.cn/v2/)^40^, and haplotype analysis was carried out using the method in the database. Only haplotypes found in ≥10 rice accessions were recorded. The wild rice sequence information was collected from the Rice Annotation Project database (https://ricegenome.dna.affrc.go.jp/). Haplotype information of Mini-Core germplasms collected worldwide by US Department of Agriculture^51^ were used to plot on the map, in which the 30-year average terrestrial temperature obtained from Esri (https://www.esri.com) were also plotted using ArcGIS (http://www.arcgis.com/).

### Nucleotide diversity and fixation index calculation

Nucleotide diversity of *SLG1* and surrounding regions were calculated on the Mini-Core dataset using a custom Perl script (https://github.com/oushujun/ThetaPi) with parameters ‘-missing_rate 1 -window_size 100′^52^. Fixation index (*F*_ST_) was calculated using VCFtools with 1000-bp windows and 100-bp steps on the 20-kb region and 10-kp windows and 1-kb steps on the 2-Mb region^53,54^.

### Phylogenetic analysis

The entire amino-acid sequence of SLG1 was used as the query to search for the homologous proteins in the NCBI website (https://blast.ncbi.nlm.nih.gov/Blast.cgi), and phylogenetic analysis was performed using MEGA7.0. The neighbor-joining method was used with 1000 bootstrap replications. For phylogenetic reconstruction of *SLG1*, the neighbor-joining trees were constructed using MEGA7.0 with pair-wise deletion for missing data.

### Proteomics and bioinformatics analysis

Shoots of 40 seedlings of *slg1* and WT at 2-leaf stage grown under normal conditions were sampled for protein extraction. The differentially expressed proteins were identified by using iTRAQ-based quantitative proteomics and LC-MS/MS by Shanghai OE Biotechnology Company (http://www.shoysw.cn/). In summary, 1610 proteins were quantified (FDR < 1%), and 112 differentially expressed proteins were selected for >1.2-fold change between *slg1* and WT. The annotation of the differentially expressed proteins between *slg1* and WT was based on the GO annotations of molecular function (http://www.geneontology.org/).

Comparison of thiolase domain was performed between SLG1 and the known thiolase domain containing proteins of AtCTU2 from *Arabidopsis thaliana* (UniProt: O65628), ScCTU2 from *Saccharomyces cerevisiae* (UniProt: P53923), and HsCTU2 from *Homo sapiens* (UniProt: Q2VPK5). Sequence alignment was conducted on the website of ClustalW (https://www.genome.jp/tools-bin/clustalw).

## Supplementary information

Supplementary Information

Description of Additional Supplementary Files

Supplementary Data 1

Supplementary Data 2

Supplementary Data 3

## Data Availability

Data supporting the findings of this work are available within the paper and its Supplementary Information files. A reporting summary for this Article is available as a Supplementary Information file. The datasets and plant materials generated and analyzed during the current study are available from the corresponding author upon request. The mass spectrometry proteomics data have been deposited to the iProX repositories with the dataset identifier IPX0002504000. Source data are provided with this paper.

## Source data

Source Data
