# Supplementary material for: Natural variations of SLG1 confer high-temperature tolerance in indica rice
Source: Nat Commun. 2020 Oct 28;11:5441. doi: 10.1038/s41467-020-19320-9 (PMC7595236; doi:10.1038/s41467-020-19320-9)
Supplement: Supplementary file 1 — Supplementary Information [file 41467_2020_19320_MOESM1_ESM.pdf]

Natural variations of *SLG1* confer high-temperature tolerance in *indica* rice

Xu *et al.*

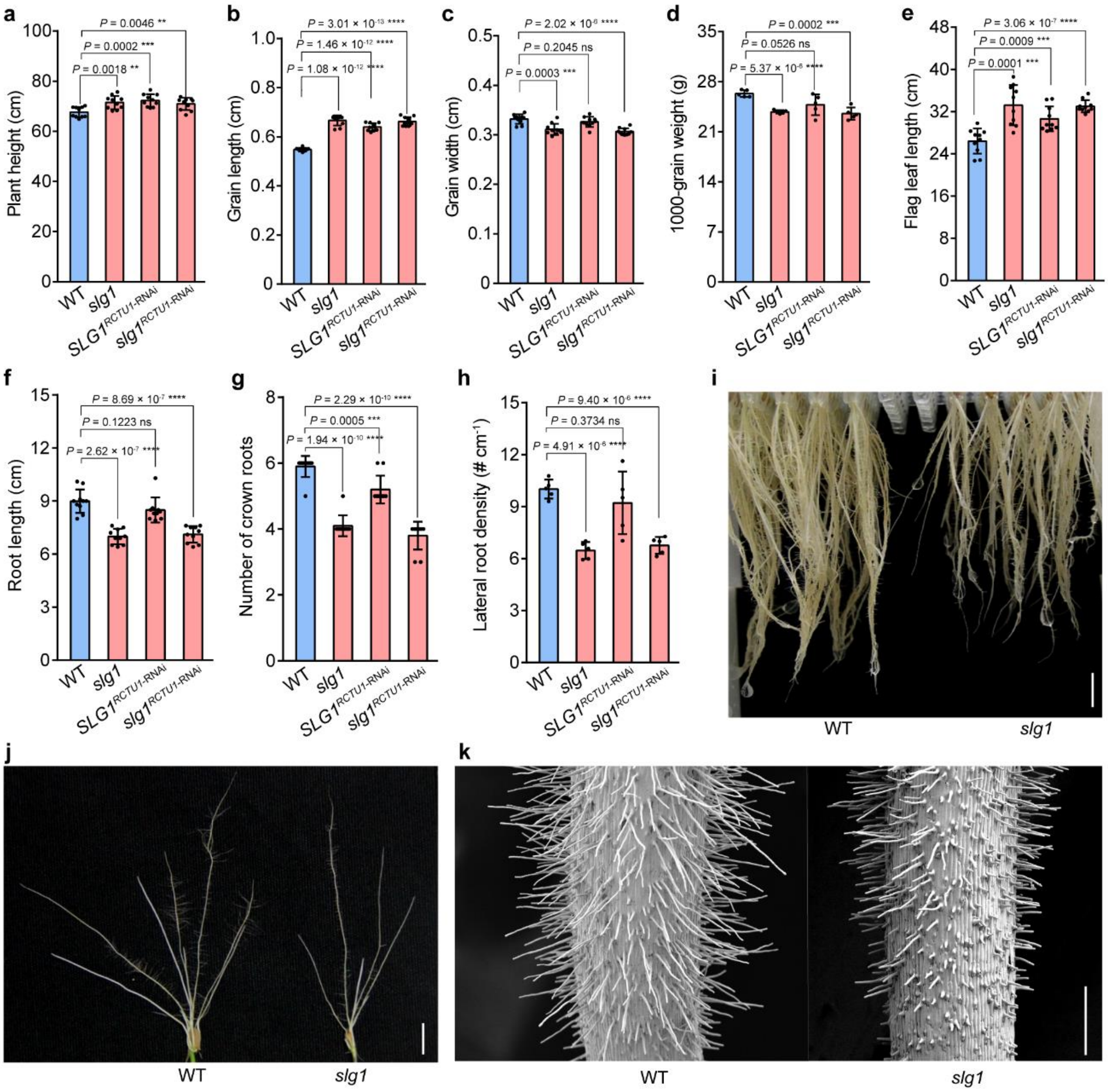

Supplementary Figure 1. *SLG1* and *RCTU1* show similar effects on multiple developmental processes.

a-h Comparison of plant height (a), grain length (b), grain width (c), 1,000-grain weight (d), flag leaf length (e), root length (f), number of crown roots (g) and lateral root density (h) among WT, *slg1*, *SLG1*$^{RCTU1\text{-RNAi}}$, and *slg1*$^{RCTU1\text{-RNAi}}$ plants. For f-h, 10-day-old seedlings were sampled. Data are means ± SD (for a-c and e-g, n = 10 plants; for d and h, n = 5 plants). **: $P < 0.01$, ***: $P < 0.001$, ****: $P < 0.0001$, ns: no significant difference, two-tailed Student's *t*-test.

i-k Abnormal root architecture in the *slg1* mutant. Bars = 1 cm (i, j) and 500 µm (k). The experiments in (i-k) were repeated three times with similar results.

The source data underlying Supplementary Fig. 1a-h are provided as a Source Data file.

a

```
WT genomic:   CTTTACAGCTATTATTACCATCTGCAGGAAGAGAACCCTTCTC
slg1 genomic: CTTTACAGCTATTATTACCATCTGCAAGAAGAGAACCCTTCTC
CDS:          ---------------------------GAAGAGAACCCTTCTC
```

b

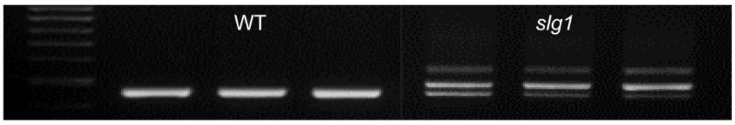

c

```
   WT: 301  PSNGINSLVASFIKRLR--------------------EENPSREHTIVRTAQKLKPFSFNKF   342
 MT-1: 301  PSNGINSLVASFIKRLRLFLLIVWRTTHLYSYYYHLQEENPSREHTIVRTAQKLKPFSFNKF   362
 MT-2: 301  PSNGINSLVASFIKRLR---------------------------------------------   317
 MT-3: 301  PSNGINSLVASFIKRLR---------------------------------------------   317
 MT-4: 301  PSNGINSLVASFIKRLR---------------------------------------------   317

   WT: 343  SADGYHDFLPSRLRPKFQKFDSDESTFSEILCLMCGSPFSESELQNLESTKHKAQKKIDLYT   404
 MT-1: 363  SADGYHDFLPSRLRPKFQKFDSDESTFSEILCLMCGSPFSESELQNLESTKHKAQKKIDLYT   424
 MT-2: 318  ------------------KFDSDESTFSEILCLMCGSPFSESELQNLESTKHKAQKKIDLYT   361
 MT-3:      --------------------------------------------------------------
 MT-4:      --------------------------------------------------------------

   WT: 405  AHCCQSCYFQILPAGENLNEHFFSLLPKLWTGKMDTISDSHSLLRDQIEEYLLEENDDGN   464
 MT-1: 425  AHCCQSCYFQILPAGENLNEHFFSLLPKLWTGKMDTISDSHSLLRDQIEEYLLEENDDGN   484
 MT-2: 362  AHCCQSCYFQILPAGENLNEHFFSLLPKLWTGKMDTISDSHSLLRDQIEEYLLEENDDGN   421
 MT-3: 318  VSSVLSCQLLHVKLHFTYS-----------------------------------------   336
 MT-4: 318  LLLPSARREPFSRAYHCKNCAKAQALLF--------------------------------   345
```

Supplementary Figure 2. The point mutation in *slg1* causes alternative splicing.

a The causal mutation detected by sequencing. A single nucleotide substitution (G to A) at the splicing acceptor site of the fifth intron of *LOC_Os12g39840* occurred in the *slg1* mutant.

b Mis-splicing caused by the mutation shown in **a**. Multiple splicing variants were detected in *slg1*, in comparison to the single transcript in WT. The experiment was repeated three times with similar results.

c Amino-acid sequences encoded by the splicing variants in the *slg1* mutant. The nucleotide sequences of the PCR products shown in **b** were determined by sequencing, and translation was performed using the DNA analysis software (Sequencher4.5).

The source data underlying Supplementary Fig. 2b is provided as a Source Data file.

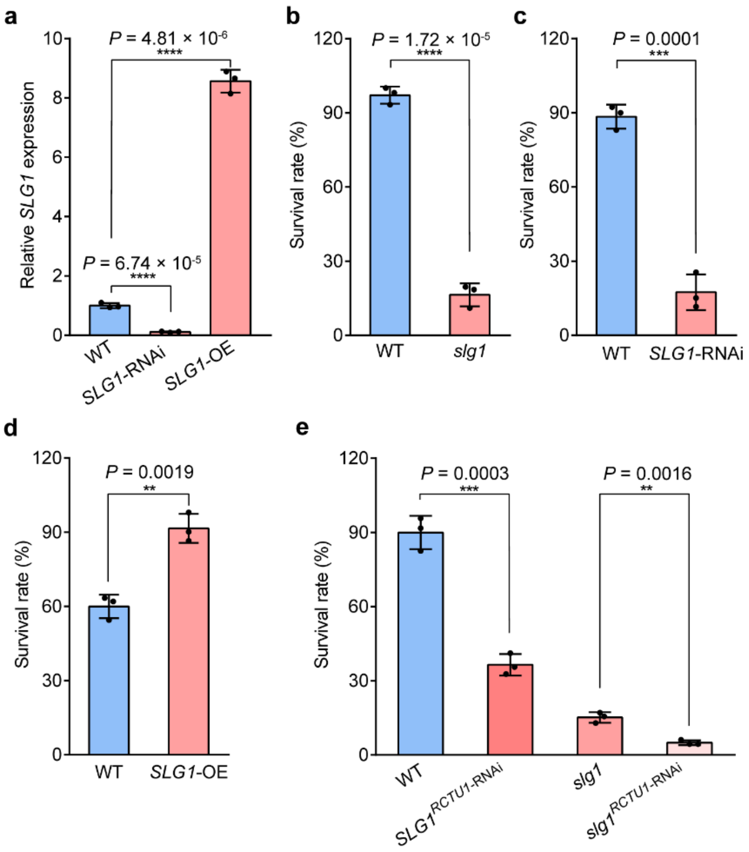

Supplementary Figure 3. Comparison of thermotolerance among WT, *slg1*, and related transgenic plants.

a Relative *SLG1* expression in WT, *SLG1*-RNAi, and *SLG1*-OE plants. Total RNA was extracted from the shoots of 2-leaf-stage seedlings grown under normal conditions, and the expression levels were determined by qPCR. The transcript level was normalized against WT, which was set to 1. *Actin* was used as the internal control. Data are means ± SD (n = 3 biological replicates).

b-e Statistical analysis of seedling survival rate after high-temperature treatment. The survival rate was determined after treatment at 45°C for 44 h (b, c, e) or 56 h (d) and recovered under normal conditions for 10 days. Data are means ± SD (n = 3 biological replicates, with 40-56 seedlings per biological replicate). **: $P < 0.01$, ***: $P < 0.001$, ****: $P < 0.0001$, two-tailed Student's *t*-test.

Source data are provided as a Source Data file.

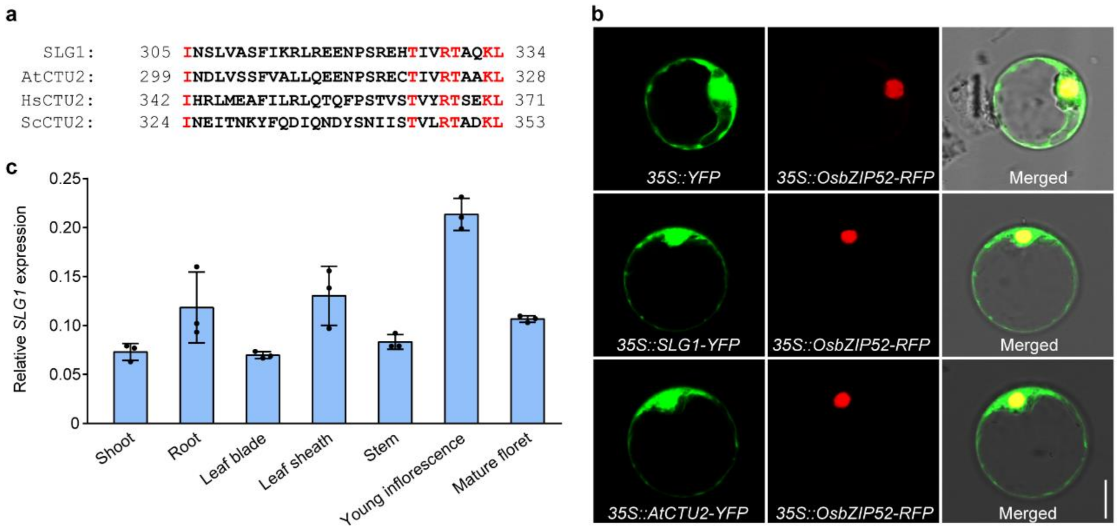

Supplementary Figure 4. Subcellular localization of SLG1 and expression pattern of *SLG1*.

a Thiolase domain comparison of SLG1 and its homologous proteins from *Arabidopsis thaliana*, *Homo sapiens* and *Saccharomyces cerevisiae*. Red letters indicate conserved amino acids.

b SLG1 and AtCTU2 are localized in both nucleus and cytoplasm. OsbZIP52 was used as the nuclear localization marker. Bar = 10 μm. The experiment was repeated three times with similar results.

c Tissue-specific expression of *SLG1*. Shoots and roots were taken from 14-day-old seedlings, and flag leaf blade and leaf sheath, stem, and mature floret were taken from plants at the flowering stage. Young inflorescences of about 1 cm in length were sampled from plants at the booting stage. The expression levels were determined by qPCR. *Actin* was used as the internal control. Data are means ± SD (n = 3 biological replicates).

The source data underlying Supplementary Fig. 4c is provided as a Source Data file.

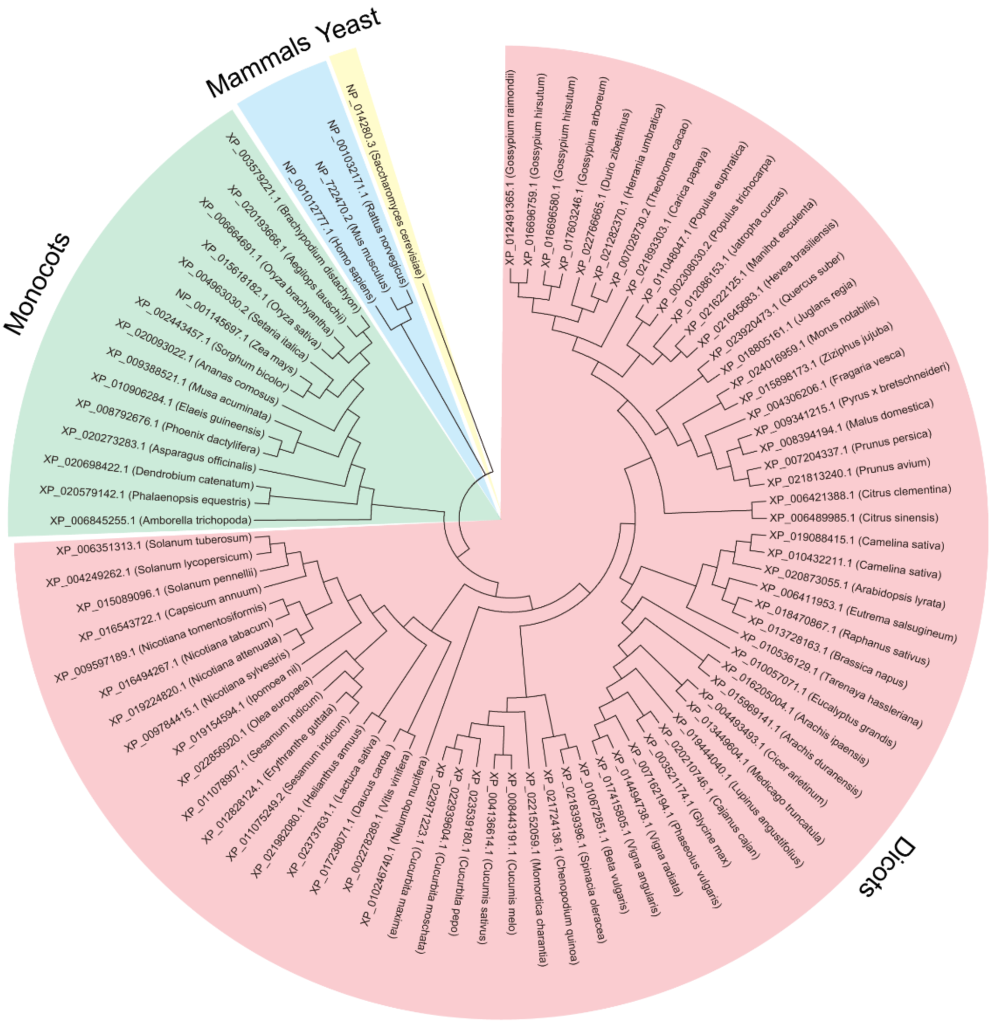

Supplementary Figure 5. Phylogenetic tree of SLG1 homologs.

The sequences of SLG1 homologs from various organisms were obtained from NCBI database searches (https://blast.ncbi.nlm.nih.gov), and phylogenetic analysis was performed using MEGA7.0. The neighbor-joining method was used with 1,000 bootstrap replications.

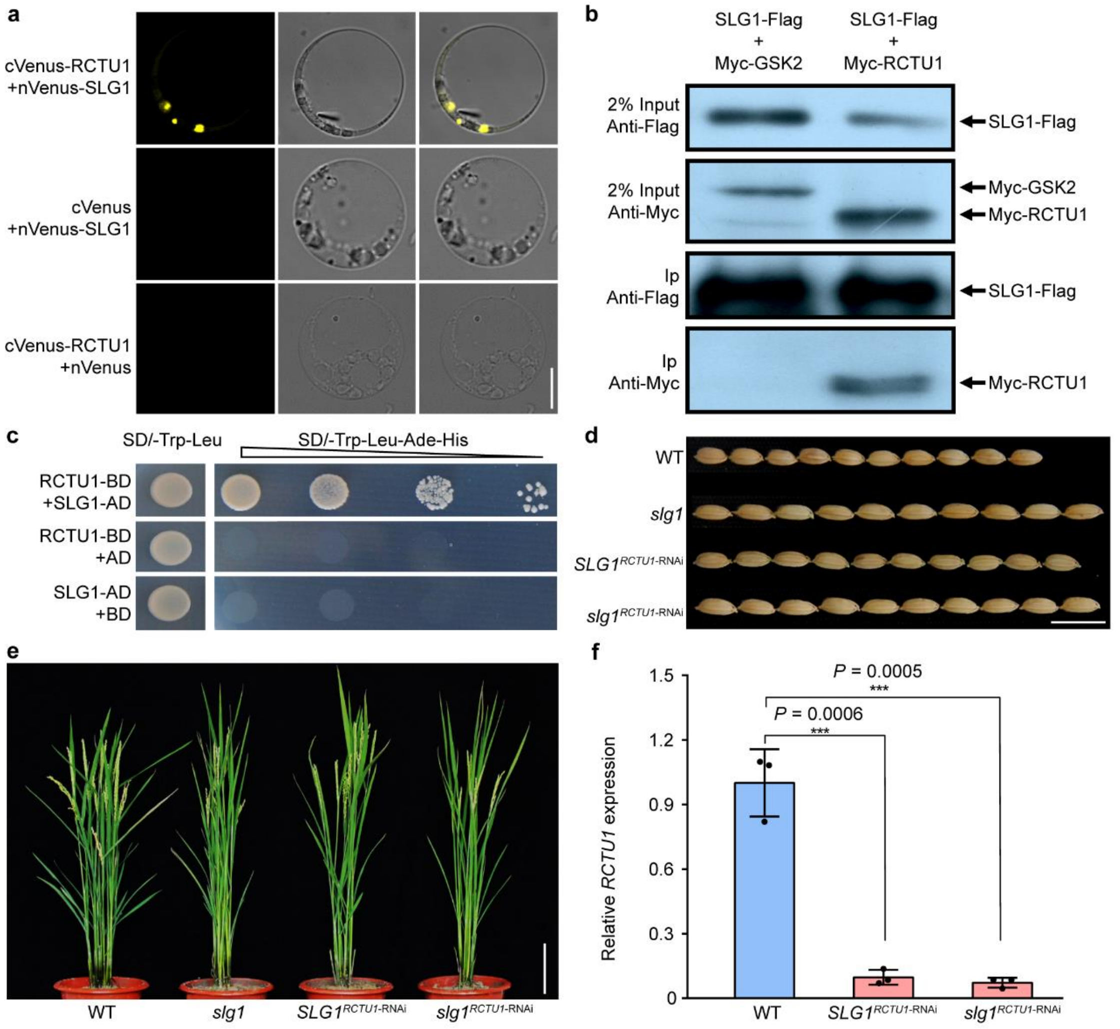

Supplementary Figure 6. *SLG1* and *RCTU1* function in the same genetic pathway.

a-c Interaction assay between SLG1 and RCTU1. The formation of SLG1-RCTU1 complex was confirmed by BiFC (a), co-immunoprecipitation (b), and yeast two-hybrid assay (c). Myc-GSK2 was used as the control (b). Bar = 10 μm (a). The experiments in (a, c) were repeated three times with similar results.

d, e Phenotypic comparison of grain length (d) and whole plant (e) among WT, *slg1*, $SLG1^{RCTU1\text{-RNAi}}$, and $slg1^{RCTU1\text{-RNAi}}$ plants. Bars = 1 cm (d) and 10 cm (e).

f Expression analysis of *RCTU1*. Total RNA was extracted from the shoots of 2-leaf-stage seedlings grown under normal conditions, and the expression levels were determined by qPCR. The transcript level was normalized against WT, which was set to 1. *Actin* was used as the internal control. Data are means ± SD (n = 3 biological replicates). ***: $P < 0.001$, two-tailed Student's *t*-test.

The source data underlying Supplementary Fig. 6b, 6c, and 6f are provided as a Source Data file.

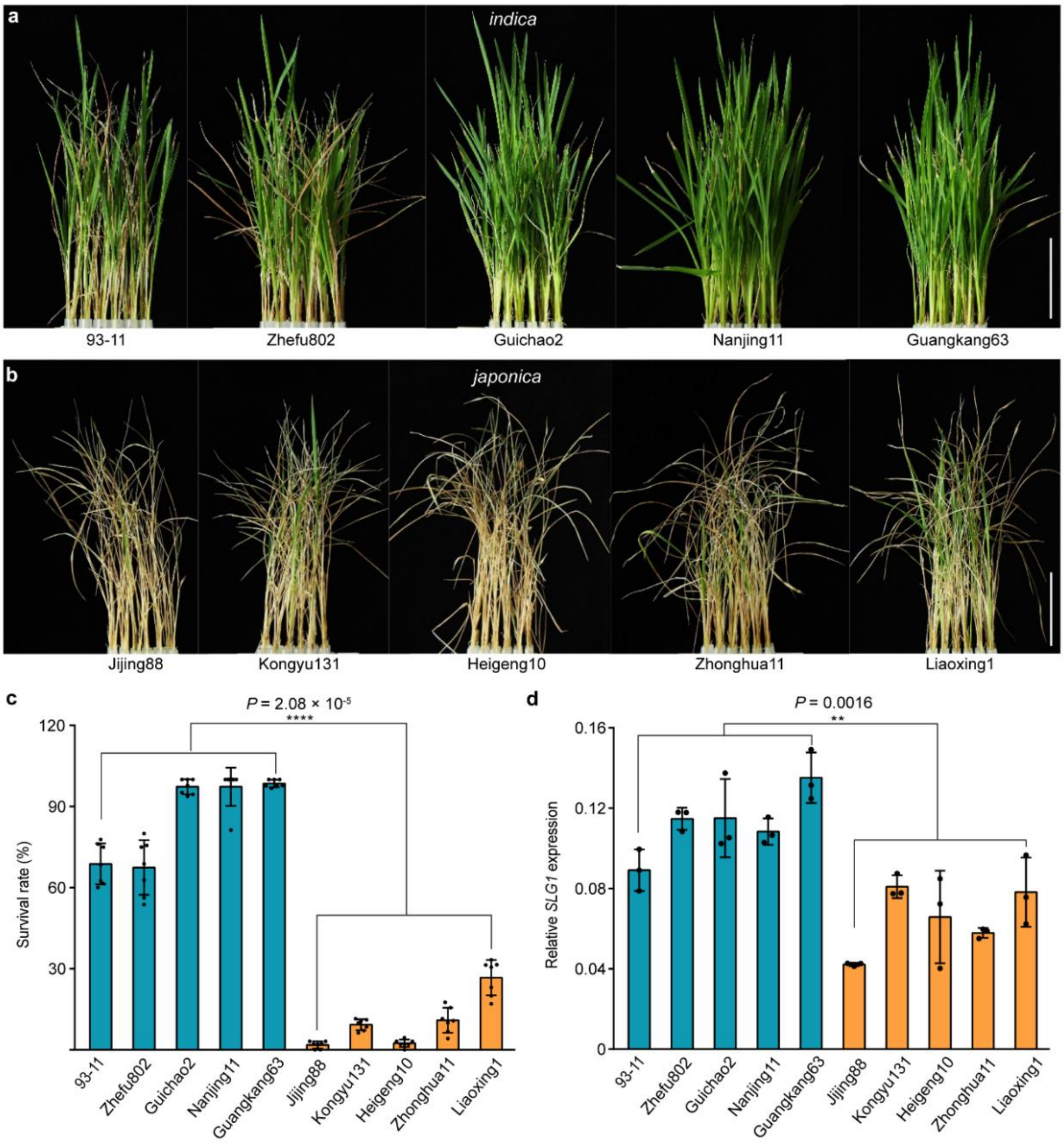

Supplementary Figure 7. The *indica* subspecies show strong thermotolerance and high *SLG1* expression level.

a-c Comparison of thermotolerance between representative *indica* and *japonica* cultivars. Seedlings of 2-leaf stage were treated at 45°C for 72 h and recovered under normal conditions for 10 days. Data are means ± SD (n = 7 biological replicates, with 32-48 seedlings per biological replicate). ****: $P < 0.0001$, two-tailed Student's *t*-test. Bars = 5 cm.

d Expression analysis of *SLG1*. Total RNA was extracted from the shoots of 2-leaf-stage seedlings under normal grown conditions, and the expression levels were determined by qPCR. *Actin* was used as the internal control. Data are means ± SD (n = 3 biological replicates). **: $P < 0.01$, two-tailed Student's *t*-test.

The source data underlying Supplementary Fig. 7c and 7d are provided as a Source Data file.

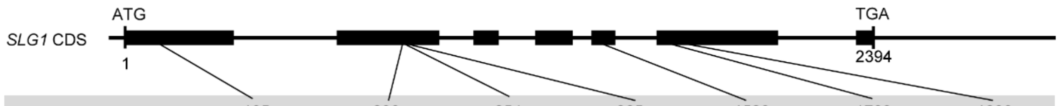

	105	839	854	865	1520	1763	1839
Nipponbare	G	A	G	C	C	C	T
93-11	T	G	A	T	T	A	G
NERICA3	G	A	G	C	C	C	T
Kasalath	T	G	A	T	C	C	G

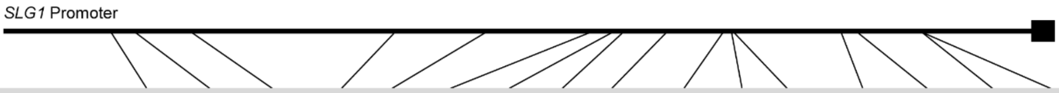

	-1396	-1353	-1271	-942	-804	-641	-616	-587	-512	-413	-402	-400	-279	-252	-164	-160
Nipponbare	C	A	AT	-	-	A	-	A	-	A	A	C	-	A	A	A
93-11	C	G	-	-	-	G	A	T	TCA<br>CTA<br>ACT<br>TC	G	A	T	CATA<br>TACA<br>TATT<br>TTCT<br>AT	C	T	-
NERICA3	C	A	AT	-	-	A	-	A	-	A	A	C	-	C	A	A
Kasalath	T	A	-	T	TT	G	-	A	TCA<br>CTA<br>ACT<br>TC	G	G	C	CATA<br>TACA<br>TATT<br>TTCT<br>AT	C	A	-

Supplementary Figure 8. *SLG1* mutations among Nipponbare, 93-11, NERICA3, and Kasalath.

Genomic sequence of *SLG1* was compared among the four cultivars representing *temperate japonica* (Nipponbare), *indica* (93-11), *tropical japonica* (NERICA3), and *aus* (Kasalath) rice subpopulations, respectively. Multiple SNPs and InDels were presented in both promoter and coding regions of the *SLG1* gene among these cultivars.

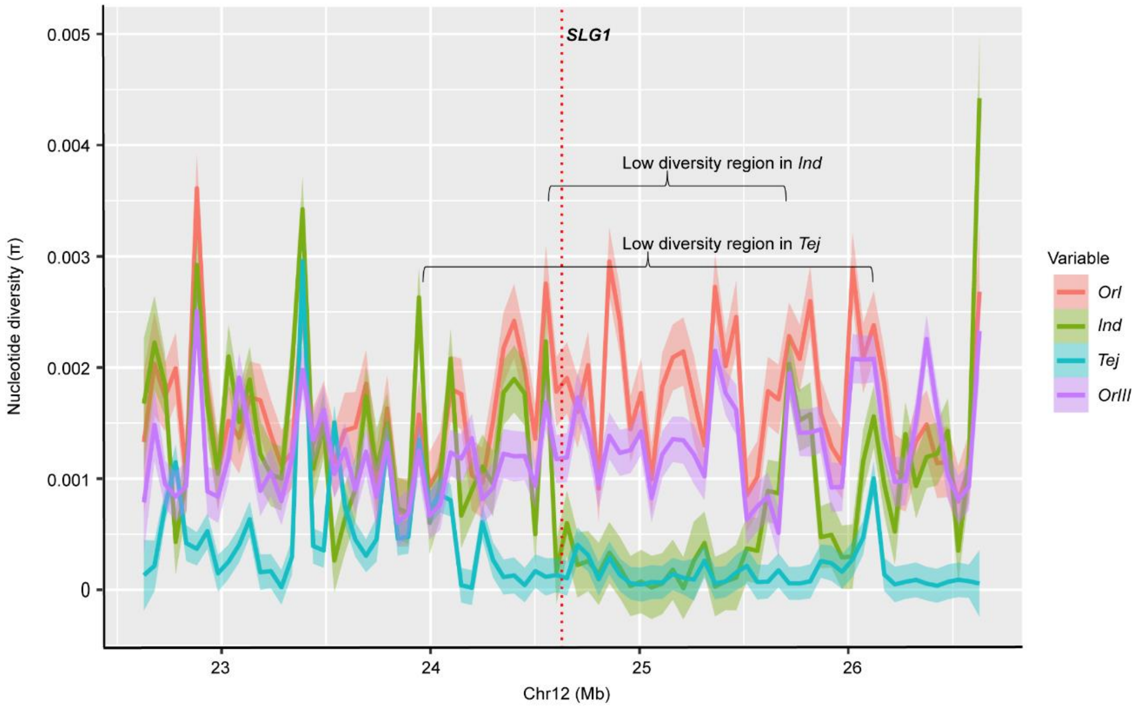

Supplementary Figure 9. Nucleotide diversities of *SLG1* in the rice subpopulations.

The X-axis denotes a 4-Mb region centered on *SLG1* and the Y-axis indicates average nucleotide diversity (π) values. The red dotted line is the position of the *SLG1* gene. Brackets outline low diversity regions of the *indica* and *temperate japonica* populations. Shades of each line outlines 95% confidence intervals given by the generalized additive mode (gam) smoothing, and the solid line is estimated by formula = y~s(x). Source data is provided as a Source Data file.

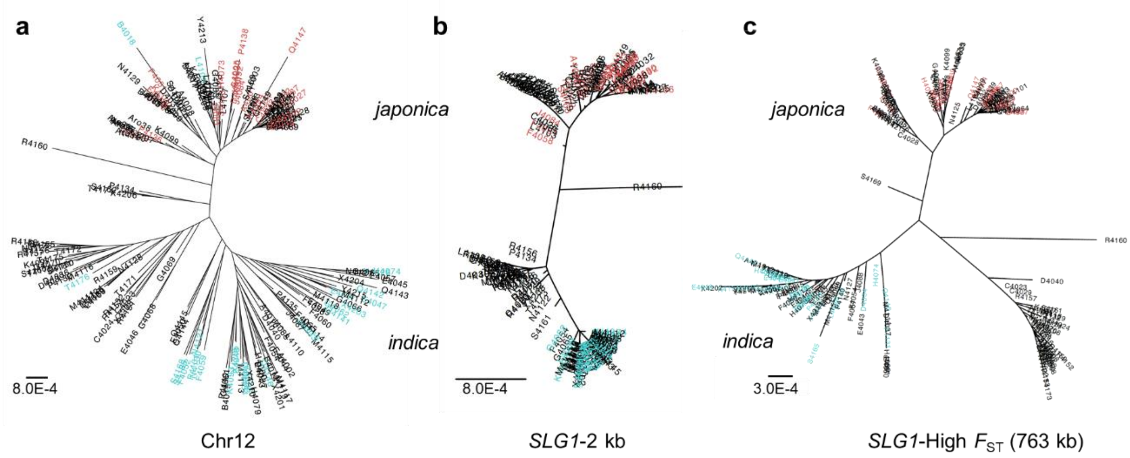

Supplementary Figure 10. Phylogenetic reconstruction of *SLG1*.

a The neighbor-joining tree based on 22-kb random SNPs on chromosome 12.

b The neighbor-joining tree based on *SLG1* and 2-kb flanking regions.

c The neighbor-joining tree based on *SLG1* and 763-kb high fixation index (F_{ST}) flanking regions.

The neighbor-joining trees were constructed using MEGA7.0 with pair-wise deletion for missing data. The Mini-Core population was used in this analysis. Admixture samples (n = 56) were labeled in color and the rest were in black, and the admixture samples carrying Hap1 and Hap2 haplotypes were colored in red and green, respectively.

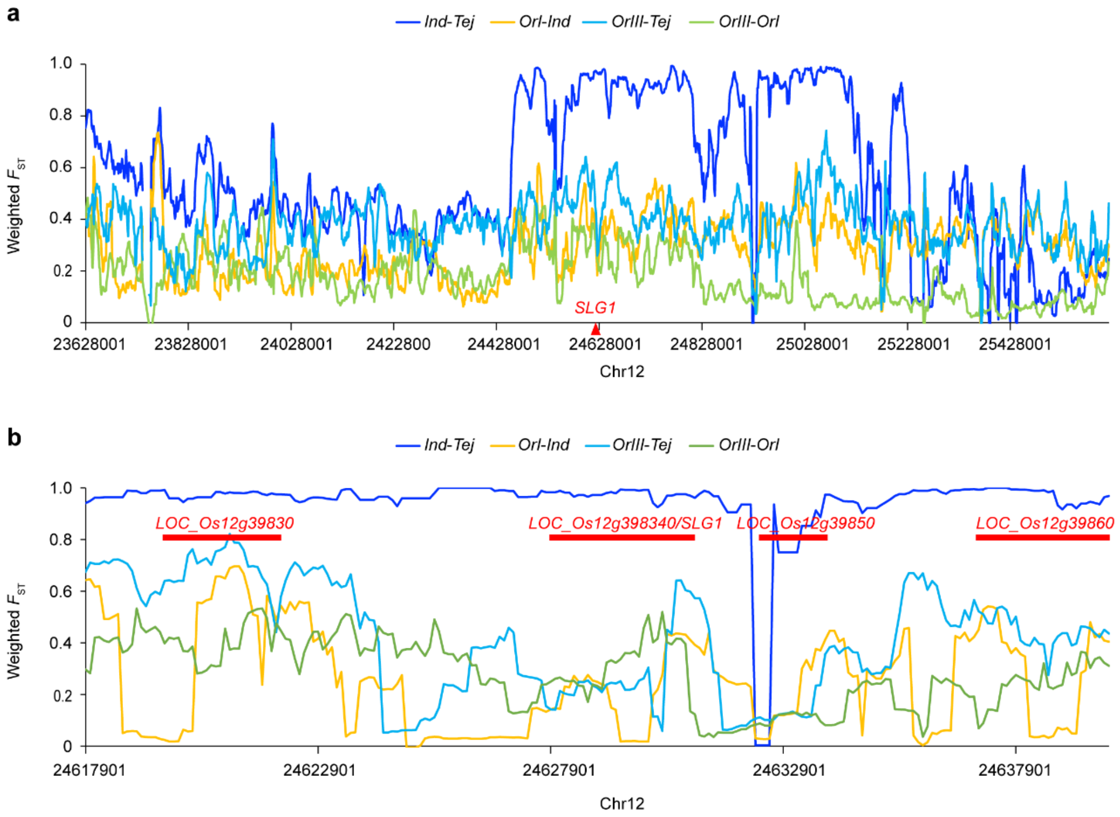

Supplementary Figure 11. Population differentiation of *SLG1*.

Y-axes are the weighted fixation index (F_{ST}) between populations. X-axes are genomic regions centered on *SLG1*.

a F_{ST} with 10-kp windows and 1-kb steps on a 2-Mb region. The *SLG1* gene is labeled with a red triangle.

b F_{ST} with 1,000-bp windows and 100-bp steps on a 20-kb region. Gene models of this region were placed accordingly.

Source data are provided as a Source Data file.

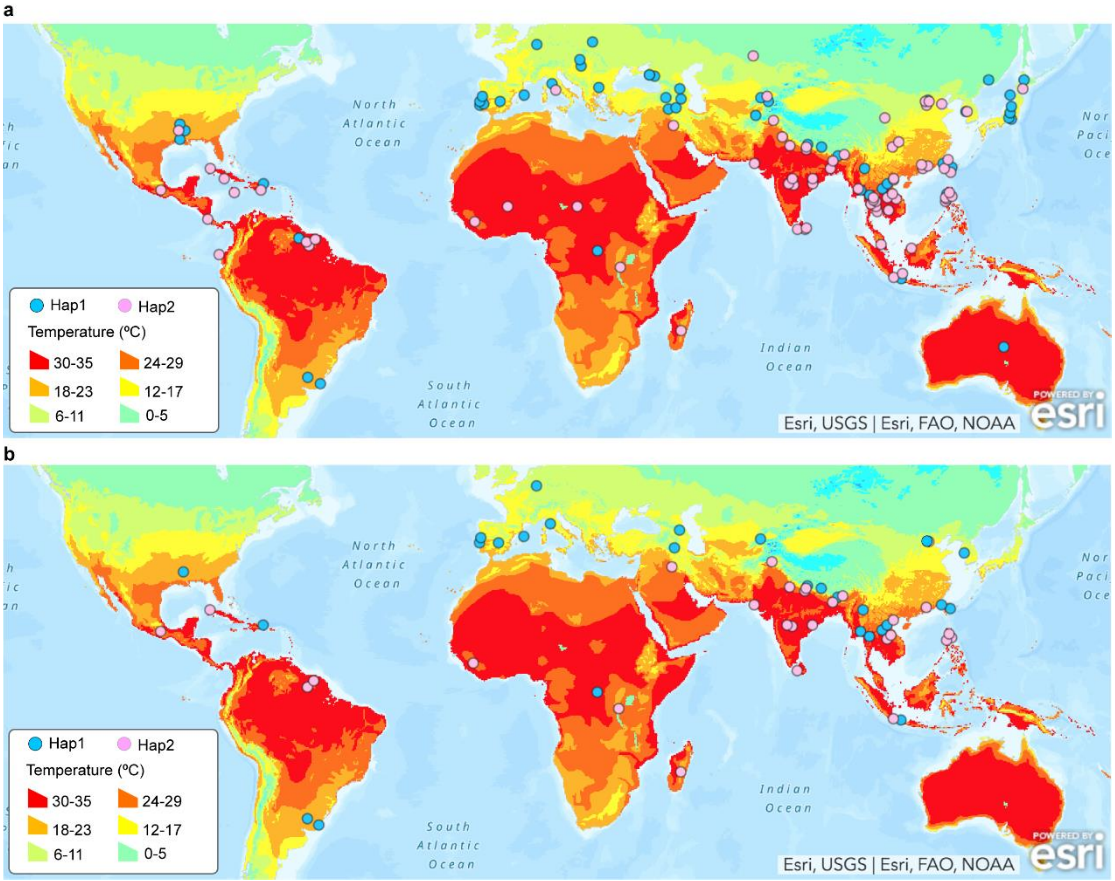

Supplementary Figure 12. Geographic distribution of *SLG1*-Hap1/Hap2 in Mini-Core germplasms.

The Hap1 and Hap2 were labeled in blue and pink dots, respectively. Each dot represents the geographic origin of a Hap1- or Hap2-carried Mini-Core germplasms (a), and a Hap1- or Hap2-carried admixture lineage in Mini-Core germplasms (b). Terrestrial surface temperatures averaged from 1960 to 1990 A.D. were shown as a heat map with scale bars in °C. Maps for sample distributions were created using ArcGIS® software from the Environmental System Research Institute, Inc. (Esri). All rights reserved.

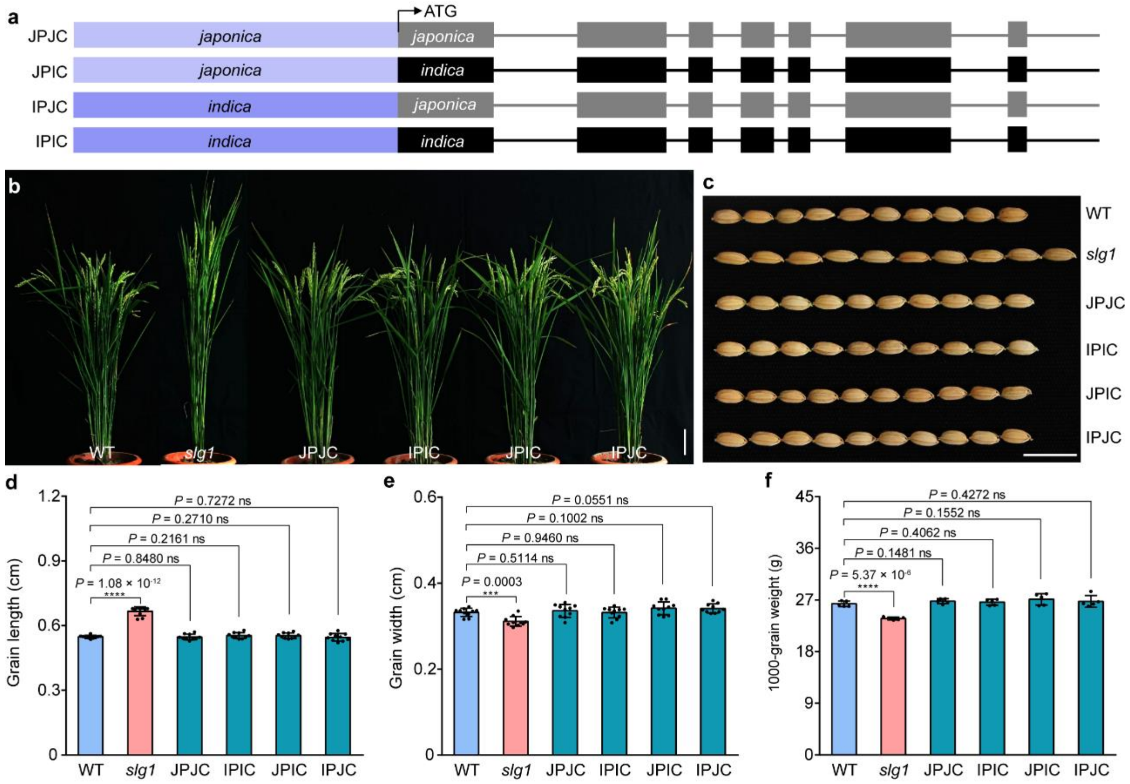

Supplementary Figure 13. Combination of the promoter and gene regions of *SLG1*^{Ind} and *SLG1*^{Tej} all could rescue the *slg1* mutant phenotype.

a Sketch of the combinatory JPJC, IPIC, JPIC, and IPJC constructs.

b, c Comparison of gross morphology and grain shape among JPJC, IPIC, JPIC, and IPJC plants. Bars = 10 cm (b) and 1 cm (c).

d-f Comparison of grain length (d), grain width (e) and 1,000-grain weight (f). Data are means ± SD (for d and e, n = 10 plants; for f, n = 5 plants). ***: $P < 0.001$, ****: $P < 0.0001$, ns: no significant difference, two-tailed Student's *t*-test.

The source data underlying Supplementary Fig. 13d-f are provided as a Source Data file.

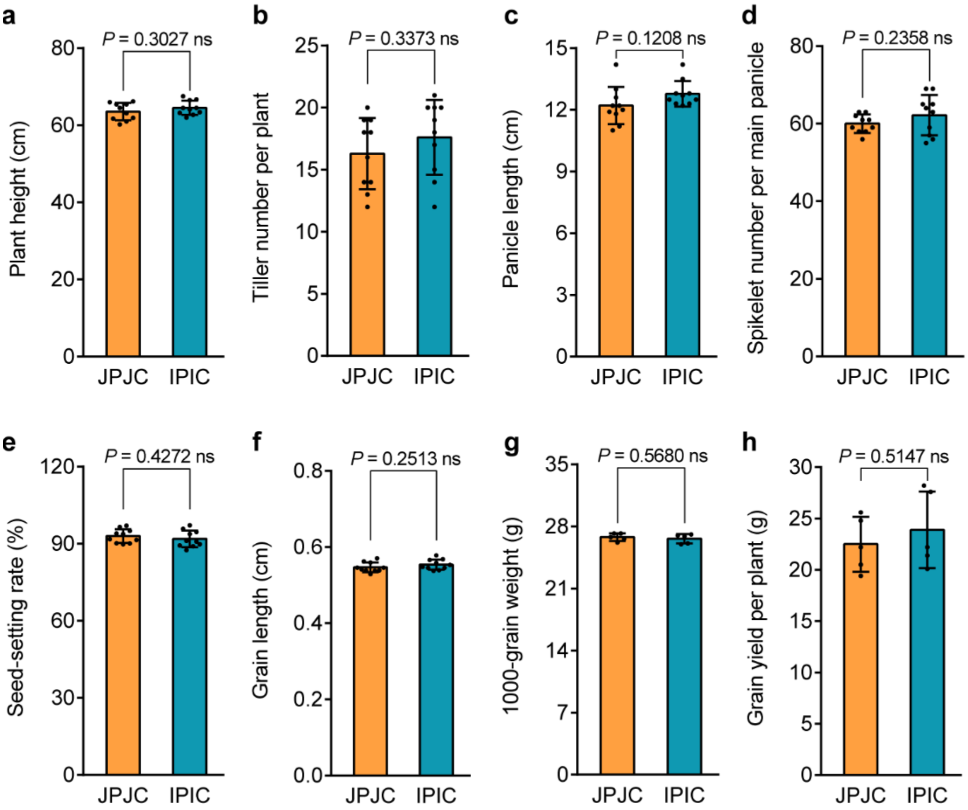

Supplementary Figure 14. *SLG1^{Ind}*- and *SLG1^{Tej}*-complemented plants show similar agronomic traits and productivity.

No significant difference was observed between JPJC and IPIC plants in plant height (a), tiller number per plant (b), panicle length (c), spikelet number per main panicle (d), seed-setting rate (e), grain length (f), 1,000-grain weight (g), and grain yield per plant (h). Data are means ± SD (for a-f, n = 10 plants; for g and h, n = 5 plants). ns: no significant difference, two-tailed Student's *t*-test. Source data are provided as a Source Data file.

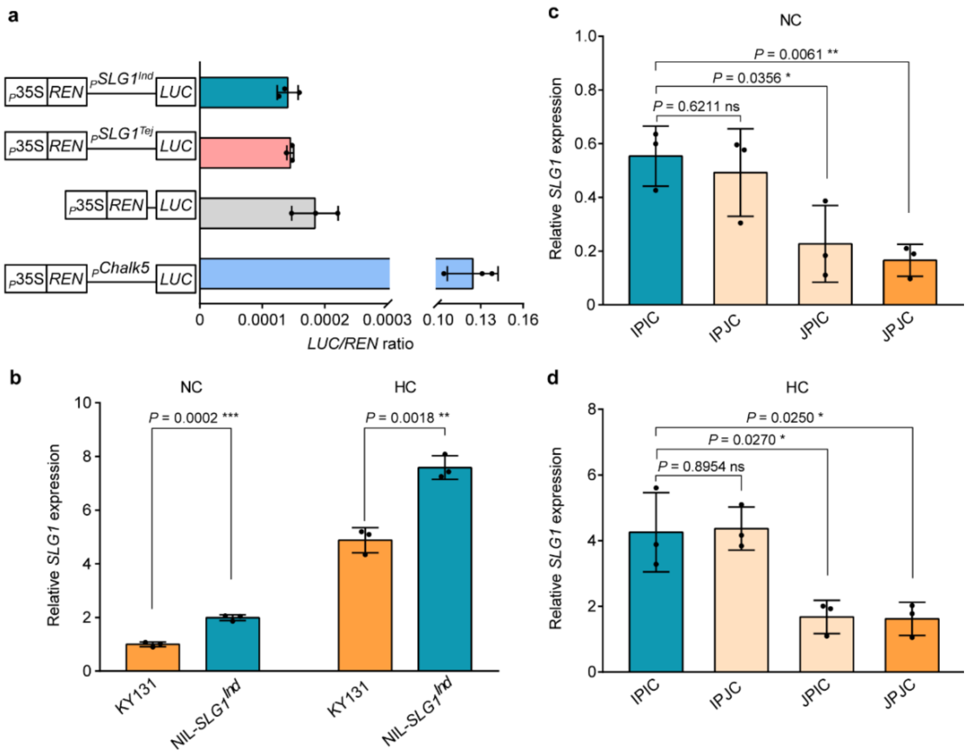

Supplementary Figure 15. Comparison of promoter activity and transcriptional response to high temperature between *SLG1*^{Ind} and *SLG1*^{Tej}.

a Promoter activity assay. $_{P}$*SLG1*^{Tej} and $_{P}$*SLG1*^{Ind} indicate the promoters of Hap1 and Hap2, respectively. The empty vector was used as the negative control and the promoter of *Chalk5* ($_{P}$*Chalk5*) as the positive control. Data are means ± SD (n = 3 biological replicates).

b Comparison of the *SLG1* expression abundance between KY131 and NIL-*SLG1*^{Ind} under different growth conditions. Total RNA was extracted from the shoots of 2-leaf-stage seedlings grown under normal condition (NC) and high-temperature condition (HC: 45°C, 12 h), respectively, and the expression levels were determined by qPCR. NIL-*SLG1*^{Ind} indicates KY131 with *SLG1*^{Ind} allele from ZF802. The expression level of *SLG1* in KY131 under normal condition was set to 1. *Actin* was used as the internal control. Data are means ± SD (n = 3 biological replicates). **: *P* < 0.01, ***: *P* < 0.001, two-tailed Student's *t*-test.

c, d Expression analysis of *SLG1* in IPIC, IPJC, JPIC, and JPJC transgenic lines under different growth conditions. Total RNA was extracted from the shoots of 2-leaf-stage seedlings grown under normal (**c**) and heat stress (**d**; 45°C, 12 h) conditions, respectively, and the expression levels were determined by qPCR. Three independent transgenic lines were used for each of IPIC, IPJC, JPIC, and JPJC. *Actin* was used as the internal control. Data are means ± SD (n = 3 independent transgenic lines, and each transgenic line was analyzed with three biological replicates). *: *P* < 0.05, **: *P* < 0.01, ns: no significant difference, two-tailed Student's *t*-test.

Source data are provided as a Source Data file.

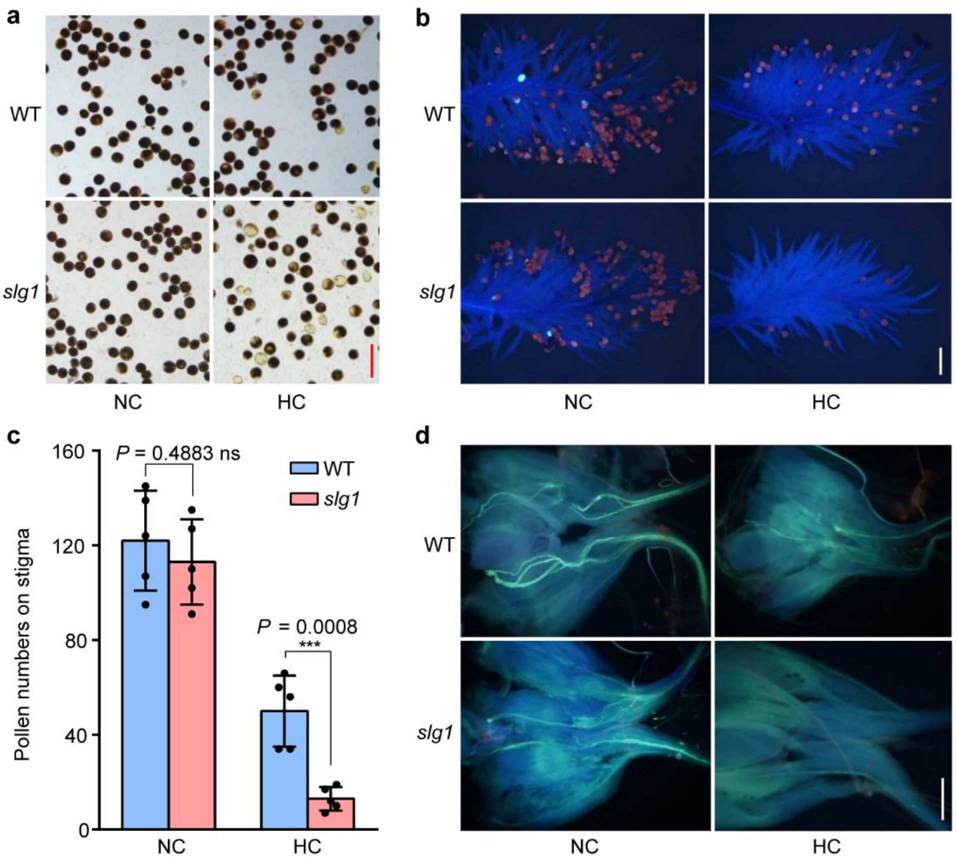

Supplementary Figure 16. The *slg1* mutant is sensitive to high-temperature stress at the reproductive stage.

a-c Comparison of the pollen fertility (a) and number of pollens in the pistil of *slg1* and WT under normal (NC) and high-temperature (HC) conditions. Pollen fertility was determined using I_2-KI staining. The number of pollens in the pistil in (b) was quantified in (c). Data are means ± SD (n = 5 pistils). ***: $P < 0.001$, ns: no significant difference, two-tailed Student's *t*-test. Bars = 100 μm.

d Comparison of pollen tube elongation on the stigma of *slg1* and WT under normal (NC) and high-temperature (HC) conditions. Bar = 100 μm. The experiments in (a, d) were repeated three times with similar results.

The source data underlying Supplementary Fig. 16c is provided as a Source Data file.

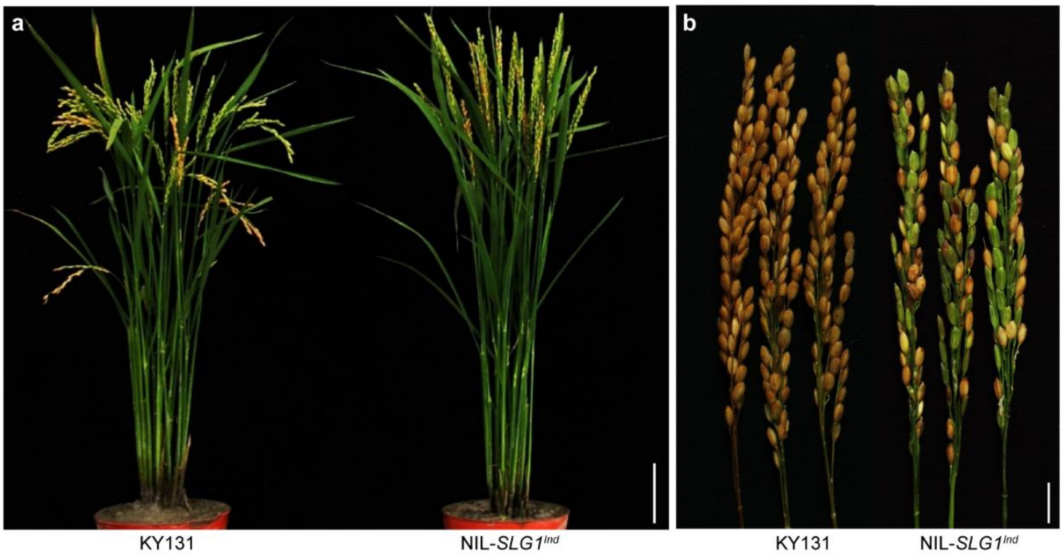

Supplementary Figure 17. Comparison of seed setting between NIL-*SLG1*^{Ind} and the recipient KY131.

The sharply reduced fertility in the NIL-*SLG1*^{Ind} plants was indicated. Bars = 10 cm (a) and 1 cm (b).

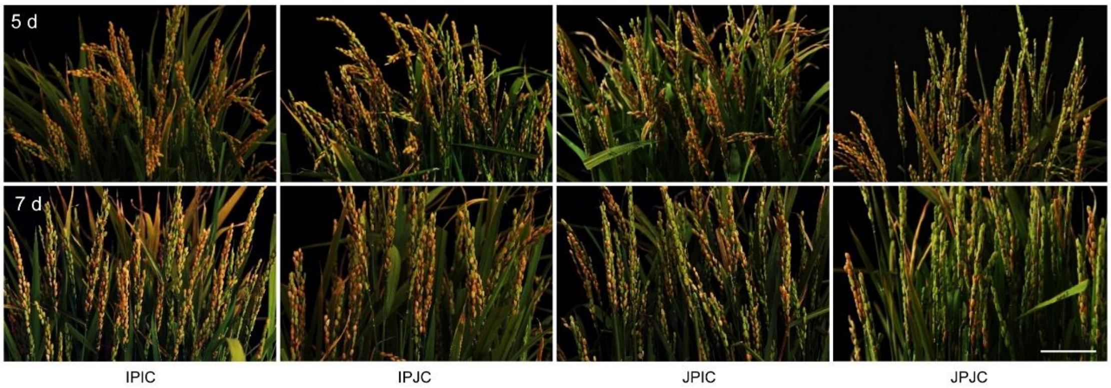

Supplementary Figure 18. ***SLG1*^{Ind}-complemented plants show strong thermotolerance at the reproductive stage.**

Homozygous IPIC, IPJC, JPIC, and JPJC lines were treated with 14 h light (40°C) / 10 h dark (31°C) for 5 days and 7 days, respectively, and recovered under normal growth conditions until seed maturation. Bar = 5 cm.

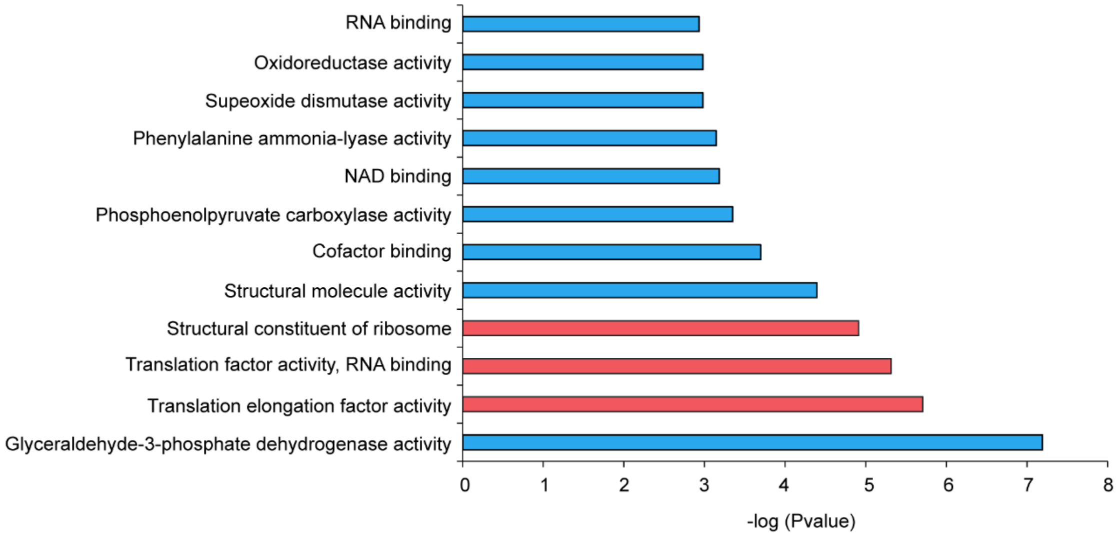

Supplementary Figure 19. Annotation of the differentially expressed proteins between *slg1* and WT based on their predicted molecular functions.

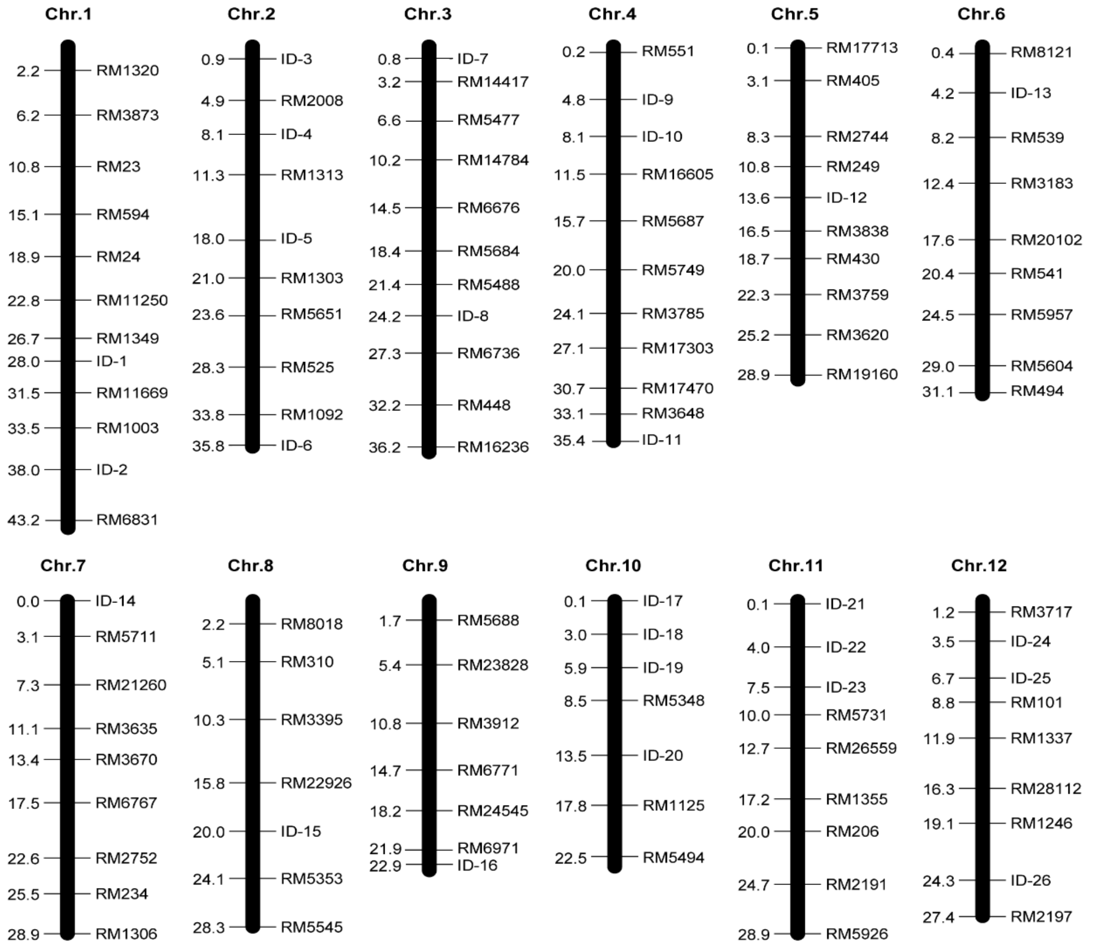

Supplementary Figure 20. Whole-genome markers used for construction of near isogenic lines.

Polymorphic markers between the two parents of KY131 and ZF802 were firstly screened in the SSR marker library (https://archive.gramene.org/markers/), and additional InDel markers were further designed based on the available information (https://ricegenome.dna.affrc.go.jp/).

Supplementary Table 1. Nucleotide diversity of *SLG1* and its flanking regions in different rice subpopulations.

Population	Downstream	*SLG1*	Upstream	Mean (flanking region)
Tej	0.000194[a*]	0.000073[a]	0.000152[a]	0.000173
Trj	0.000524[a]	0.000541[a]	0.000560[a]	0.000542
Ind	0.000705[a]	0.000098[a]	0.001239[b]	0.000972
Aus	0.000210[a]	0.000073[ab]	0.001058[b]	0.000634
Or-I	0.003284[a]	0.002261[a]	0.003513[a]	0.003398
Or-II	0.003458[a]	0.005659[b]	0.004857[b]	0.004158
Or-III	0.003519[a]	0.004024[a]	0.004296[a]	0.003908
Wild	0.004129[a]	0.004098[a]	0.004688[a]	0.004408

	Downstream	*SLG1*	Upstream
π(*Or-III*) / π(*Tej*)	18.14	55.00	28.26
π(*Or-III*) / π(*Trj*)	6.72	7.43	7.67
π(*Or-I*) / π(*Ind*)	4.66	23.18	2.84
π(*Or-I*) / π(*Aus*)	15.66	30.90	3.32

* The different superscript letters following the values represent significant difference at 1%. two-tailed Student's *t*-test. Source data are provided as a Source Data file.
