# Supplementary material for: Natural variations of SLG1 confer high-temperature tolerance in indica rice
Source: Nat Commun. 2020 Oct 28;11:5441. doi: 10.1038/s41467-020-19320-9 (PMC7595236; doi:10.1038/s41467-020-19320-9)
Supplement: Supplementary file 2 — Description of Additional Supplementary Files [file 41467_2020_19320_MOESM2_ESM.pdf]

Description of Additional Supplementary Files

Supplementary Data 1

Sequence variations of SLG1 in wild rice accessions

Supplementary Data 2

Differentially expressed proteins in the slg1 mutant

Supplementary Data 3

Primers used in this study.
